# Foodomics in Diabetes Management: A New Approach

**DOI:** 10.1002/fsn3.71021

**Published:** 2025-09-28

**Authors:** Sammra Maqsood, Muhammad Tayyab Arshad, Ali Ikram, Hatem A. Al‐Aoh, Kodjo Théodore Gnedeka

**Affiliations:** ^1^ National Institute of Food Science and Technology University of Agriculture Faisalabad Faisalabad Pakistan; ^2^ Functional Food and Nutrition Program, Center of Excellence in Functional Foods and Gastronomy, Faculty of Agro‐Industry Prince of Songkla University Songkhla Thailand; ^3^ University Institute of Food Science and Technology The University of Lahore Lahore Pakistan; ^4^ Analytical Chemistry Research Laboratory, Department of Chemistry, Faculty of Science University of Tabuk Tabuk Saudi Arabia; ^5^ Togo Laboratory: Applied Agricultural Economics Research Team (ERE2A) University of Lomé Lomé Togo

**Keywords:** genomics, metabolomics, omics, proteomics

## Abstract

Critical information regarding the interactions among food components, human metabolism, and disease is contained in foodomics, an interdisciplinary field that bridges food science with contemporary omics technologies (genomics, proteomics, metabolomics, and lipidomics). In order to gain a better understanding of the metabolic dysregulation in type 2 diabetes mellitus (T2DM), foodomics examines bioactive compounds derived from food (e.g., polyphenols, fibers, and lipids) alongside host molecular responses. For the enhancement of glycemic control and the prevention of diabetes‐related complications, the current study is concerned with how foodomics enables personalized dietary interventions that are aligned with one's metabolic and genetic characteristics. We investigate deeper into the role of the gut microbiota in T2DM progress and how foodomics‐informed methodologies, such as metabolomics and metagenomics, can be functional to discover treatments intended at the microbiota. In addition, we discover the prospective that functional foods enriched with bioactive elements, comprising β‐glucans and flavonoids, may influence metabolic processes in diabetes. In addition, foodomics improves food safety by recognizing conceivable diabetes‐causing contaminants (endocrine disruptors). Foodomics has incredible potential for improving precision nutrition in the prevention and treatment of T2DM, though experiments in data integration and standardization are present. Through the integration of dietary concepts, molecular biology, and clinical consequences, this method offers revolutionary strategies towards metabolic wellness.

## Graphical abstract

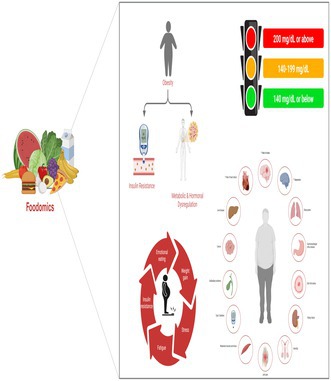

## 1 Introduction

The new multidisciplinary science of foodomics brings together the science of food with cutting‐edge omics sciences such as genomics, proteomics, metabolomics, and lipidomics to explore the intricate molecular interactions between food components and disease (Balkir et al. 2021). Foodomics, as defined by Ibáñez and Cifuentes (2014), offers valuable information on nutrition‐related disorders, especially T2DM, through the study of bioactive food constituents like lipids, dietary fibers, and polyphenols and their corresponding biological effects. Personalized diet therapy is within sight, with this strategy having the capacity to systematically describe how an individual's nutrients affect metabolic networks, gene expression, and the microbiota composition of the gut (Capozzi and Bordoni 2013).

The assembly of several omics information is where foodomics is built. Genomics, as elucidated by Fitipaldi et al. (2018), classifies genetic variants (e.g., TCF7L2 and PPARγ polymorphisms) that affect food metabolism and human responsiveness to dietary variations. In order to examine protein expression patterns and find indicators of diabetes‐caused β‐cell dysfunction, proteomics employs two‐dimensional electrophoresis and mass spectrometry (MS) (Andjelković and Josić 2018). Metabolomics works with nuclear magnetic resonance spectroscopy and LC–MS to profile ceramides and branched‐chain amino acids as metabolites and relate their dysregulation with insulin resistance, leading to Backman et al. (2019).

Lipidomics examines lipid species such as oxylipins and sphingomyelins and uncovers their roles in inflammation and the expansion of diabetes, according to Wu et al. (2021). Through the grouping of food science with cutting‐edge omics technologies comprising proteomics, metabolomics, lipidomics, and genomics, the newly emerged multidisciplinary science of foodomics is revolutionizing our understanding of the intricate interfaces among food and human health (Balkir et al. 2021). Using the newfangled vision, we are capable of analyzing biological systems at a molecular level, as well as food nutritional value and conformation. According to Ibáñez and Cifuentes (2014), foodomics differs from classical nutritional science in that it takes a systems‐level approach that can explain the complex associations between food and health effects. Genetic studies in foodomics aim to find how different people's genes affect their reactions to different elements of foodstuff. This is particularly so when conducting the investigation on SNPs in genes like TCF7L2 and PPARγ, which influence nutrient metabolism (Fitipaldi et al. 2018).

Proteomics approaches systematically examine the protein structure of foods and their interface with the human biological system, as stated by Andjelković and Josić (2018). This makes us comprehend the manner in which food acts and whether or not it is an allergen. Metabolomics refers to the branch of science scrutinizing how diet has effects on biochemical pathways through applying analysis platforms that measure and distinguish among metabolites employing nuclear magnetic resonance (NMR) spectroscopy and liquid chromatography‐mass spectrometry (LC–MS) (Valdés et al. 2021).

Lipidomics is a subdiscipline of metabolomics that uses advanced mass spectrometry approaches to classify and understand the functions of lipid molecular species in metabolic control and disease onset (Wu et al. 2021). These omics approaches are complementary to aid in elucidating the molecular mechanisms by which food distresses human physiology. There are numerous main areas of nutrition and health science that can be appreciated in foodomics.

Zheng and Chen (2014) elucidate that cutting‐edge analytical technologies with high throughput are vital for food safety because they disclose food adulterants, confirm product authenticity, and define harmful contaminants that could affect metabolic health. The capability of foodomics to analyze the interfaces among diet and gut microbiota is significant as it enables the demonstration of how microbial metabolism of food content affects metabolic pathways in the host that are responsible for preventing disease (He et al. 2021). This capability has fueled the developments in precision nutrition, as emphasized by Vimaleswaran et al. (2015). Here, omics information is used to deliver individualized dietary recommendations on the basis of the individual's metabolic and genetic profile. T2D is a multifactorial metabolic syndrome with topographies of insulin resistance and impaired glucose metabolism; these technologies have special prospects in the treatment of T2D.

Chronic hyperglycemia, insulin insensitivity, and dyslipidemia are the pathophysiology of this metabolic syndrome. Nephropathy, neuropathy, and cardiovascular illness are significant consequences that have a tendency to obfuscate (Jaacks et al. 2016). In the case of understaffed healthcare institutions and inadequate patient access to therapy, the treatment of type 2 diabetes is further complex (Godman et al. 2020). Diabetes care is an expensive endeavor, with yearly healthcare costs projected at hundreds of billions of dollars internationally (Seuring et al. 2015). In numerous respects, foodomics offers novel solutions to these tasks. These approaches enable earlier diagnosis and more precise treatments by recognizing molecular markers of disease risk and progression (Fitipaldi et al. 2018). For erecting individualized dietary plans that also consider metabolic differences, it is important to exhaustively elucidate the nutrient‐gene interactions (Vimaleswaran et al. 2015).

Foodomics also sheds light on how certain nutrients such as polyphenols, fiber, and fatty acids affect glucose homeostasis metabolic networks (Bordoni and Capozzi 2014). In conclusion, technology helps us to better comprehend the relations of our diet with our microbiome, which switches inflammation and insulin sensitivity. From this, nutritional medicines targeting definite bacteria can then be shaped (He et al. 2021). There is confidence that T2DM anticipation and management can be revolutionized through foodomics data in clinical and public health settings. It is now conceivable to recognize new food bioactive composites, which will more effectively control metabolism (Ren and Li 2019). Rendering to Idehen et al. (2020), there can be making of functional foods that can address precise metabolic needs through the application of exhaustive profiling methods. An example contains β‐glucan‐enriched breakfast cereals for glucose control.

Foodomics delivers a scientific foundation for tailored dietary references that can enhance glycemic control and minimize diabetes‐associated complications, which is perhaps the most significant real‐world application of nutritional science (Bordoni and Capozzi 2014). But, there are still obstacles to being overwhelmed in terms of standardizing methodology, assimilating multi‐omics data, and translating investigation results into the clinical environment (Hasin et al. 2017). The amended characterization of diet‐health relations will become possible with the help of new technologies like single‐cell omics policies and machine learning intended to overcome these limits (Caratti et al. 2024). The field of foodomics will continue to grow in use in diabetes investigation and management as it continues to develop. This will open up new channels for the insight and regulation of molecular relationships among nutrition and metabolic health (Li et al. 2023). The state of the art of foodomics technologies, their applications to T2DM investigation, and how these approaches can revolutionize dietary means of averting and treating diabetes are all deliberated in this review.

In the current review article, we deliberate on how foodomics has established over time and how foodomics is used in the management of T2DM through precision nutrition. This study places of interest the newest technologies intricate in foodomics, such as genomics, proteomics, metabolomics, and lipidomics, in order to further elucidate the relationship between food and health and the effect of this relationship on T2DM. This review is about how foodomics facilitates the understanding of the etiology, pathogenesis, and treatment of diabetes based on multi‐omics methods. This review also efforts to show the promise of personalized nutritional guidance based on an individual's own genetic and metabolic profile, which could lead to better glycaemic control and fewer diabetes‐related complications. Concerns about foodomics in hazard assessment, food safety, and the determination of bioactive substances that can be exploited to treat T2DM are also addressed in this study, which is concentrated on functional foods.

## 2 Foodomics Technologies and Techniques

The study of food constituents and the metabolic outcomes that ensue is recognized as foodomics, and it predominantly comes into play when conducting diabetes investigations; subsequently, it involves a diversity of cutting‐edge analytical platforms. Genomic approaches such as metagenomics are joining the fray with three fundamental methods: chromatography, mass spectrometry (MS), and nuclear magnetic resonance (NMR) spectroscopy as obligatory (Chelliah et al. 2022).

**FIGURE 1:**
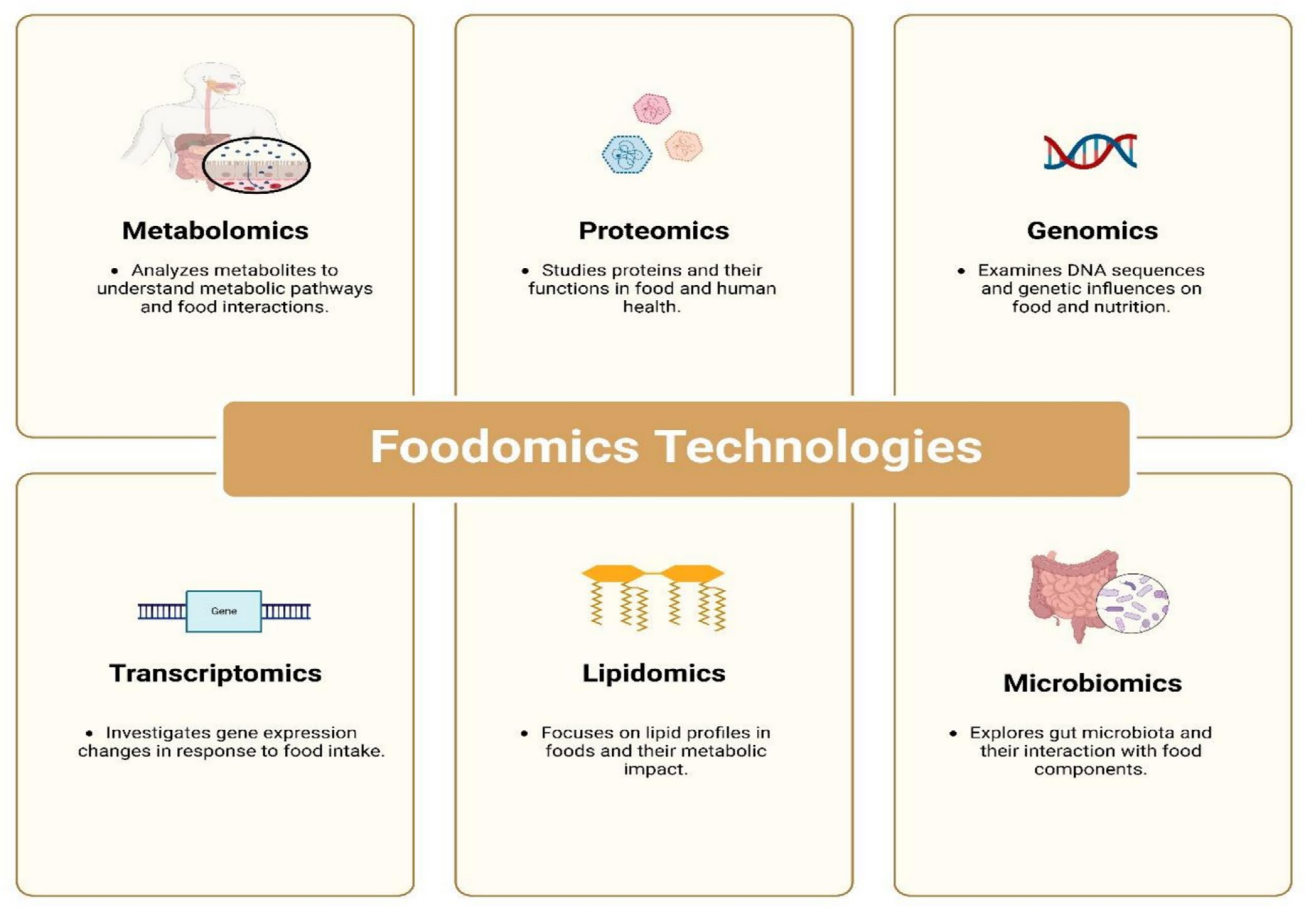
Foodomics technologies.

Though each procedure has certain benefits, together these shed light on the molecular procedures of T2D in an overarching way. Chromatography can distinguish intricate food matrices, nuclear magnetic resonance (NMR) offers non‐destructive structural characterization, and mass spectrometry (MS) proposes exquisite sensitivity for detecting and quantifying biomolecules (Gallo and Ferranti 2016). These technologies permit the discovery of biomarkers and improved insight into T2DM pathophysiology, along with other omics approaches such as proteomics and genomics (Figure 1). Personalized diet treatment can be focused on foodomics to control diabetes, as established in studies (de Toro‐Martín et al. 2017). Figure 1 shows the foodomics technologies.

### 2.1 Mass Spectrometry (MS)

Because of its distinct capability to analyze many biomolecules, mass spectrometry has become a significant analytical technique in foodomics studies. The technique can be used to classify what molecules are in a sample by ionizing them, unraveling them based on their mass‐to‐charge ratio and eventually detecting them, as clarified by Herrero et al. (2012). Among the numerous applications of MS to diabetes investigation, metabolomics and lipidomics methodologies that identify metabolic dysregulation stand out particularly (Chung et al. 2018). With MS, Herrero et al. (2012) positively diagnosed with T2DM in its early stage by recognizing distinctive metabolic patterns reflective of the advancement of the disease. This technique is particularly suited for the examination of diabetes patients' complex metabolic modifications because of its high sensitivity and capability to detect metabolites in very low concentrations (Maqsood et al. 2025).

By lipidomics analysis accomplished by MS, Wu et al. (2021) presented that this competence could be utilized to uncover certain lipid abnormalities in T2DM, leading to novel insights into lipid metabolism dysregulation. The protein expression profiles associated with diabetes, i.e., in insulin resistance pathways, have been recognized by MS‐based proteomics policies, as exemplified by Andjelković and Josić (2018) and Meng Jia et al. (2019). The consequence of MS in driving our molecular understanding of T2DM is demonstrated by these applications.

### 2.2 Nuclear Magnetic Resonance (NMR) Spectroscopy

Among the most significant analytical methods in foodomics, nuclear magnetic resonance spectroscopy has distinct compensations in diabetes investigation. Nuclear magnetic resonance, or NMR, is a method that determines the molecular conformation and structure by the detection of the resonance frequencies of atomic nuclei in a strong magnetic field when excited by radiofrequency pulses (Corsaro et al. 2016). Nuclear magnetic resonance (NMR) testing does not damage samples; therefore, further testing can be achieved if essential (Fan and Zhang 2019). Investigators working on diabetic‐related metabolic changes could chiefly benefit from the use of NMR because it is non‐destructive and also delivers absolute quantitation of metabolites without standards (Fan and Zhang 2019).

Using nuclear magnetic resonance (NMR), Laghi et al. (2014) could profile the metabolic variations in diabetic patients to reveal what constituents of food cause specific metabolites to shift. Canela et al. (2016) also presented that the technique has been used to confirm functional foods and supplements used as diabetes control aids. Moreover, NMR is perfect for real‐time observation of metabolic reactions to diet interference; meanwhile, it is capable of measuring complex biological samples with minimal preparation (Hatzakis 2019). Through these usages, nuclear magnetic resonance (NMR) is more and more intricate in diabetes‐related scientific and clinical studies.

### 2.3 Chromatographic Techniques

The mainstream of foodomics analyses depends on chromatographic technology, which permits the separation of constituents from mixtures. All three of the main types of liquid chromatography can contribute to diabetes investigation: gas chromatography, high‐performance liquid chromatography, and liquid chromatography (Gilbert‐López et al. 2017).

**FIGURE 2:**
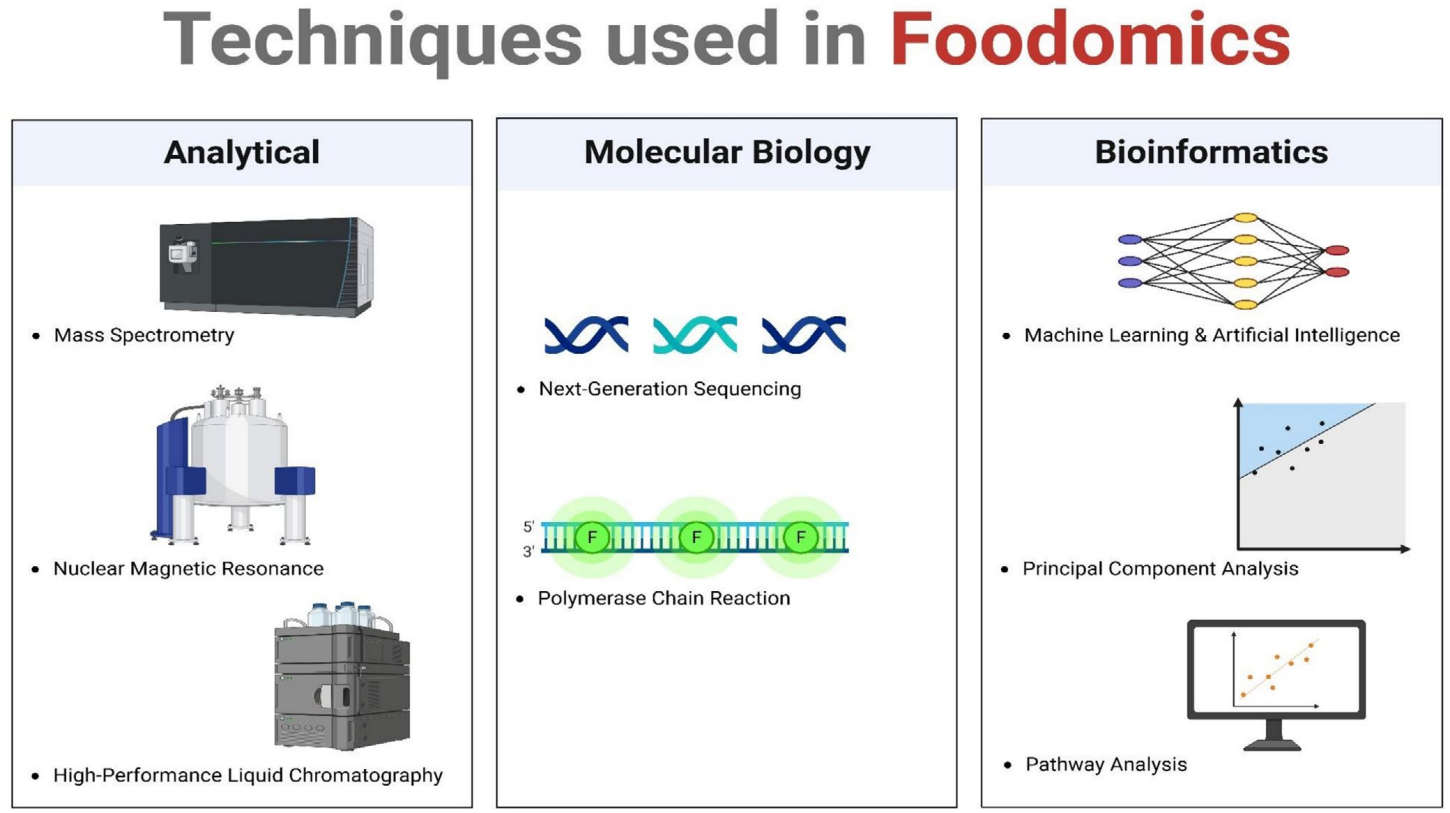
Techniques used in foodomics.

Molecular possessions such as size, charge, and polarity control which composites are separated by these approaches, which comprise differential interactions among mobile and stationary phases. Chromatography has been extremely useful in diabetes investigation to identify which bioactive food elements impact glucose metabolism, according to Ghallab et al. (2024). With LC–MS, Muguruma et al. (2022) characterized metabolic profiles of diabetic patients and recognized markers intricate in the pathogenesis of the disease. Functional foods, for instance, fiber and polyphenol‐rich foods that can be loaded with antidiabetic composites can be examined more effectively using this technique (Figure 2) (Idehen et al. 2020). Figure 2 shows the techniques used in foodomics.

Chromatography and mass spectrometry, in the view of Cajka (2024), facilitate extensive metabolomic profiling of food that could assist in T2DM treatment. González‐Sálamo et al. (2021) advise that these applications demonstrate the potential of chromatographic approaches for the explanation of causes of disease and novel nutritional treatments.

## 3 Integration of Technologies in Multi‐Omics Approaches

A merger of these examination platforms with other omics methods creates holistic multi‐omics approaches in diabetes studies, and foodomics actually comes into its own at that point (Damarov et al. 2024). Investigators can now merge metabolic data with proteomic and genetic information, conferring to Eddy et al. (2020), giving an enhanced knowledge of type 2 diabetes pathophysiology. This plan was exemplified by Valdés et al. (2021) and Herrero et al. (2012), who used an amalgamation of NMR‐based metabolomics and MS‐based proteomics to regulate biomarkers that may predict the onset of T2DM. In addition to furthering our knowledge of the disease, these high‐throughput studies pave the way for more customized diet plans based on one's genetic profile (Ďásková et al. 2023).

Andjelković et al. (2017) established the prospective of foodomics technology for the fast documentation of biomarkers and therapeutic targets for the facilitation of personalized treatment regimens. Barnett et al. (2015) demonstrate how technology synergies are altering diabetes care with newfangled molecular knowledge of personalized nutrition and functional food production. According to Liu et al. (2022), the future of diabetes investigation lies in the convergence of numerous omics technologies. This will bring on novel avenues for studying and addressing this multifactorial metabolic disease. Multi‐omics analysis progress has unveiled new genetic and metabolic indicators that can potentially change the direction of diabetes management (Li et al. 2024). Alongside, these consequences have generated concern about the role that environmental influences, such as endocrine disruptors, play in the disease's development (Dagar et al. 2023).

### 3.1 Integration of Genomics, Metabolomics, and Proteomics

The most common metabolic abnormalities associated with T2DM are insulin resistance and beta‐cell dysfunction. T2DM has been better clarified due to recent enhancements in multi‐omics, that is, genomics, proteomics, and metabolomics. By using these technologies, new biomarkers and prospective therapeutic targets to treat diabetes are recognized due to more insights into molecular alterations (Arshad et al. 2025).

#### 3.1.1 Genomics in T2DM

Examining gene expression outlines and disease‐related mutations is the key to genomics. Augmented risk of T2DM has been related to numerous genetic variants, but most meaningfully with those affecting insulin signaling or glucose and lipid homeostasis regulation. Genomic investigation making risk prediction possible by recognizing disease‐associated single nucleotide polymorphisms (SNPs) is currently available (Hasin et al. 2017). As far as control of insulin secretion is concerned, TCF7L2 plays a central role and is among the principal T2DM genetic susceptibility loci (Wang et al. 2021).

In addition to enlightening pathogenic genes, genomics also delivers insight into epigenetic alterations like DNA methylation and histone modification. Modifications can change gene expression without changing the DNA sequence. The reason for T2DM is strongly reliant on environmental factors such as stress, diet, and exercise, all of which affect these epigenetic modifications (Yousri et al. 2023). Investigators can better grasp the multifaceted interaction of hereditary predisposition and environmental influences in the pathogenesis of T2DM by joining genomic data with other omics layers (Liu et al. 2022).

#### 3.1.2 Metabolomics in T2DM

Metabolomics refers to the study of small molecules or metabolites. These are cellular biochemical impressions. This technique fills the gap directly between phenotype and genotype by enlightening the biochemical changes in the body as and when they take place in real time. Dyslipidemia, insulin resistance, and diminished glucose metabolism are characteristic metabolic profiles in type 2 diabetics (Zhang et al. 2025). According to Meneilly and Elliott (1999), metabolomic profiling may aid in identifying the biomarkers that can be used to track the onset, course, and effectiveness of a disease.

Rendering to Cajka (2024), insulin resistance and metabolic deregulation in people with T2DM can result from augmented levels of branched‐chain amino acids (BCAAs) and other metabolites such as acylcarnitines. To classify and measure thousands of metabolites in biofluids such as plasma, urine, or tissue samples, analytical platforms such as LC–MS and NMR spectroscopy are extensively applied in T2DM metabolomics (Muguruma et al. 2022). To better elucidate the mechanisms of disease, investigators tend to combine omics data with metabolomic data to examine biomolecule correlations (Gallo and Ferranti 2016).

#### 3.1.3 Proteomics in T2DM

The investigation area in relation to proteins, their interfaces, post‐translational modifications, and functional activities is called proteomics. T2DM has been interrelated with protein expression alterations connected to inflammation, insulin signaling, and glucose metabolism, as revealed by proteomic studies. For illustration, Wang et al. (2021) inform that insulin receptor substrates (IRS) and protein tyrosine phosphatases (PTPs) moderate cytokine secretion and other proteins responsible for beta‐cell damage and insulin resistance. At an early point, diagnostic and prognostic biomarkers, as well as upcoming therapeutic targets, might be resolved with proteomics. The capability to inspect complicated mixtures of proteins at high throughput has been made possible through progressions in mass spectrometry‐based proteomics (Wang et al. 2013).

To detect disease‐associated proteins and paths, technologies such as LC–MS/MS allow the discovery and quantification of proteins from biological samples (Picariello et al. 2012). For instance, Tiwari et al. (2023) specified that biomarkers of diabetic nephropathy and cardiovascular disease, two common comorbidities of T2DM, have been recognized by plasma protein profiling.

#### 3.1.4 Multi‐Omics Approaches in T2DM

Azam et al. (2019) represent that molecular mechanisms accountable for T2DM are more understandable by assimilating genomes, metabolomics, and proteomics. Tayanloo‐Beik et al. (2021) show that the fusion of multi‐omics data accounts for the multifaceted relationship between genes, proteins, and metabolites of the disease. According to Wang et al. (2021)'s investigation, this policy has the potential to transform our knowledge of inflammation, lipid metabolism, and mitochondrial function, all of which underpin insulin resistance and beta‐cell dysfunction. Assimilating data from multiple layers of omics allows investigators to build comprehensive models of disease causation encompassing environmental, metabolic, and genetic influences. In addition to guiding tailored treatment protocols, these models are also used to control early biomarkers and track the disease progression (Anwardeen et al. 2024).

Precision medicine, in which the customized genetic and biochemical profile of a patient is employed to improve treatment outcomes for T2DM, is an additional area where multi‐omics technologies are gaining traction (Wang et al. 2022). In order to have a better knowledge of the relationship between metabolic disorders like T2DM and food, investigators are trusting in foodomics, which is the science based on omics technology to study food and nutrition. As an instance, González‐Sálamo et al. (2021) conducted proteome and metabolomic analysis to study the influence of a Mediterranean diet on insulin sensitivity. Metabolite levels, particularly of amino acids and polyunsaturated fatty acids, were significantly changed, and these changes were related to lower inflammation and better insulin sensitivity they exposed. We can see here how precise food groups affect T2D‐related metabolic activities.

An additional study that studied the effect of fiber on metabolic health and gut microbiota among people with T2DM was Hosomi et al. (2022). The investigators utilized multi‐omics strategies to demonstrate that higher fiber intake led to greater numbers of advantageous bacteria like *Blautia wexlerae* , which in turn improved glucose metabolism and insulin sensitivity. This imitates how diet modification affects the metabolic well‐being of diabetics. A similar study by Sha et al. (2020) discovered the influence of a specific food on diabetic nephropathy, a serious T2DM problem. The investigators noticed biomarkers linked to glucose metabolism and kidney function by assimilating proteome and metabolomic analysis. Metabolic profiles of patients were significantly changed after the intervention, as indicated by abridged inflammatory markers and enhanced kidney function. The significance of foodomics in averting and controlling diabetes is unraveled in this case. In conclusion, genetics, metabolomics, and proteomics have merged to meaningfully upsurge the understanding of T2DM metabolic derangement. These omics methods permit the creation of biomarkers, drug targets, and individualized treatments. Additionally, novel methods for nutritional interferences to improve metabolic status have been provided using foodomics, a field that could be researched in dietary‐disease correlations. Greater addition of omics technology will ease better understanding of the intricate biology of T2DM and formation of more targeted and efficacious approaches for averting and treating the condition (Table 1). Table 1 depicts the advancements in foodomics technologies and techniques for diabetes research.

**TABLE 1:** Advancements in foodomics technologies and techniques for diabetes research.

Technology	Application in diabetes research	Case studies/Examples	References
Mass spectrometry (MS)	Proteomics for biomarker discovery in T2DM; lipidomics for metabolic profiling	MS lipidomics identified phospholipid dysregulation in diabetic nephropathy. LC–MS revealed glycated hemoglobin as a T2DM biomarker	Wu et al. (2021), Agrawal et al. (2013), Xi et al. (2022)
Nuclear magnetic resonance (NMR)	Non‐invasive metabolic profiling for early T2DM detection; gut microbiota analysis	NMR detected elevated branched‐chain amino acids in prediabetes. NMR imaging revealed pancreatic β‐cell dysfunction in T1D	Hatzakis (2019), Canela et al. (2016), Corsaro et al. (2016)
Chromatography	Metabolomics of glucose/lipid profiles; food contaminant analysis (e.g., endocrine disruptors)	LC–MS linked phthalate exposure to insulin resistance. GC × GC–MS resolved dietary polyphenols' anti‐diabetic effects	Cajka (2024), Chevalier and Fénichel (2015), Montero and Herrero (2019)
Multi‐omics integration	Combines genomics, proteomics, and metabolomics for precision therapy and biomarker discovery	Integrated multi‐omics predicted metformin response in T2DM. Gut microbiota‐metabolome networks linked to T2DM	Anwardeen et al. (2024), Wigger et al. (2021), Henneke et al. (2022)
Lipidomics	Profiling lipid alterations in diabetic complications (e.g., cardiomyopathy, nephropathy)	MS lipidomics identified cardiolipin oxidation in diabetic hearts. Ceramide species correlated with insulin resistance	Li et al. (2024), Xi et al. (2022), Faulkner et al. (2020), Chen et al. (2020)
Proteomics	Protein interaction networks in β‐cell dysfunction; dietary protein impact on insulin signaling	High‐resolution MS mapped β‐cell proteome changes in T2DM. Peptidomics revealed bioactive peptides in anti‐diabetic foods	Wigger et al. (2021), Ibáñez et al. (2013)
Genomics	Identification of T2DM susceptibility loci; nutrigenomics for personalized diets	GWAS identified *TCF7L2* variants interacting with high‐carb diets. Precision nutrition for T2DM in Benin	Yousri et al. (2023), Fitipaldi et al. (2018), Alaofè et al. (2024)
Epigenomics	DNA methylation linked to insulin resistance; dietary modulation of epigenetic marks	High‐fat diet‐induced methylation changes in *PPARγ*. Epigenomic‐metabolomic integration in diabetic retinopathy	Zhou et al. (2019), Li et al. (2024).
Metabolomics	Identification of metabolic shifts (e.g., diabetic retinopathy, nephropathy)	LC–MS revealed gut microbiota‐derived metabolites (e.g., TMAO) in T2DM. NMR detected urinary fructose‐1,6‐bisphosphate in prediabetes	Li et al. (2024), Hosomi et al. (2022), Fan and Zhang (2019)
AI and machine learning	Enhances omics data integration for predictive diagnostics and personalized nutrition	AI models predicted T2DM progression using gut metagenomics. Deep learning optimized dietary interventions	Caratti et al. (2024), Vinhaes et al. (2024), Garg and Heber (2024)
Glycomics	Analysis of glycoproteins/glycans in T2DM; dietary glycans' role in gut microbiota modulation	Glycoproteomics identified *IGFBP3* glycosylation as a T2DM biomarker. Barley β‐glucans improved glycemic control	Gallo and Ferranti (2016), Idehen et al. (2020)
Nutrigenomics	Diet‐genome interactions; personalized nutrition for T2DM management	Inulin supplementation altered T2DM biomarkers via *FFAR2* modulation. *Moringa oleifera* 's anti‐diabetic effects	Ďásková et al. (2023), Chhikara et al. (2021)
Metagenomics	Gut microbiota analysis in T2DM pathophysiology; probiotic interventions	*Parasutterella* associated with fatty acid biosynthesis in obesity/T2DM. *Blautia wexlerae* improved insulin sensitivity	Zhou et al. (2019), Hosomi et al. (2022).
Systems biology	Holistic modeling of diabetes using multi‐omics; network analysis of metabolic pathways	Liver dysfunction pathways in T2DM via multi‐omics. Kidney disease classification using omics	Backman et al. (2019), Eddy et al. (2020)

## 4 Role of Foodomics in Understanding T2DM Pathophysiology

Recent insights into T2DM pathogenesis are provided by foodomics, a cross‐disciplinary science that unites proteomics, metabolomics, and genomics. With the solicitation of cutting‐edge analytical technologies, foodomics is the investigation of how metabolites, lipids, and proteins vary between patients with T2DM. In the quest to assist early therapies before clinical presentation, foodomics allows one to detect biomarkers that may lead to screening and forecasting susceptible subjects (Muguruma et al. 2022).

Foodomics has performed wonders by revealing the association between our gut bacteria and T2DM. The gut microbiome is one of the key topographies of T2DM and plays a role in insulin resistance, bodywide inflammation, and metabolism (Tayanloo‐Beik et al. 2021). By the usage of foodomics technologies, experts can discover the effects of dietary constituents on the composition and function of gut microbiota and how these variations affect the metabolic health of diabetic patients (Wu et al. 2021). With this novel knowledge comes the opportunity of tailoring dietary therapy to the patient's microbiome in hopes of averting or treating T2DM. In order to comprehend the role of the gut microbiota in the expansion and onset of T2DM, foodomics is compulsory.

### 4.1 Metabolic Changes in T2DM: A Foodomics Perspective

Chronic hyperglycemia (high blood glucose) is a feature of T2DM, with insulin resistance, reduced insulin secretion, and augmented hepatic glucose output (Lima et al. 2022). T2DM is associated with multiple metabolic impairments, comprising systemic inflammation, oxidative stress, and dyslipidemia, in the accumulation of high blood glucose levels. To generate novel diagnostic and therapeutic strategies, it is essential to understand these metabolic variations (Zhang et al. 2012). As part of the effort to better comprehend the multifaceted nature of T2DM, investigators in the multidisciplinary domain of foodomics have been exploiting high‐throughput approaches from metabolomics, lipidomics, and proteomics. In this segment, we will elucidate how foodomics assists in clarifying the role of nutrition in disease risk and progression, biomarker discovery, and detection of metabolic changes (Vanweert et al. 2022).

#### 4.1.1 Metabolic Alterations in T2DM

T2DM is an illness of glucose metabolism, as distinguished by Lima et al. (2022). Insulin resistance blocks the entry of glucose into peripheral tissues and triggers the liver to overproduce glucose. Protein and lipid metabolism are also meaningfully obstructed by the disease. Accumulation of lipids in organs other than adipose tissues, like the liver and skeletal muscle, enhances insulin resistance; elevated triglyceride and free fatty acids (FFA) levels are typical in type 2 diabetic patients (Zhang et al. 2012).

Foodomics has proven a number of biochemical alterations involved in T2DM, most importantly metabolomics (Lima et al. 2022). A snapshot of dynamic metabolism is provided by metabolomics, the comprehensive analysis of small‐molecule metabolites. Liquid chromatography‐mass spectrometry (LC–MS) and other sophisticated analytical methods have made it possible to detect and quantify such metabolic changes extremely sensitively and specifically (Vanweert et al. 2022).

#### 4.1.2 Glucose and Insulin Metabolism

Interference with insulin signaling causes an abnormality of glucose metabolism, a feature of T2DM. Resistance to insulin diminishes the peripheral tissue's capacity for glucose uptake, producing prolonged hyperglycemia (Lima et al. 2022). Metabolomic profiling with mass spectrometry has identified that people with T2DM have higher concentrations of glucose and lactate, which may indicate a change from aerobic glycolysis to oxidative phosphorylation (Zhang et al. 2025). Apart from this, insulin sensitivity has also been associated with decreased concentrations of BCAAs (Vanweert et al. 2022).

Lipid accumulation also impacts the effectiveness of insulin. Both insulin resistance and lipotoxicity are promoted by increased free fatty acids (FFAs) and certain lipid species, including ceramides (Lima et al. 2022). Foodomics, one of the fields, lipidomics, has helped determine insulin resistance and T2DM development changes in phospholipids, triglycerides, and sphingolipids in plasma and adipose tissue (Zhang et al. 2023). These lipid biomarkers identified open important avenues towards the understanding of disease etiology and targetable sites for intervention.

#### 4.1.3 Inflammation and Oxidative Stress

One of the main etiological causes of T2DM is oxidative stress, which is also accompanied by chronic low‐grade inflammation. Augmented levels of pro‐inflammatory cytokines like interleukin‐6 (IL‐6) and tumor necrosis factor‐alpha (TNF‐α) are related to the onset of insulin resistance in type 2 diabetic patients. Biomarkers of oxidative stress and inflammation in biological matrices can be efficiently monitored by means of foodomics approaches. Oxidative stress, after a mismatch between antioxidant defenses and reactive oxygen species (ROS), causes endothelial dysfunction, β‐cell apoptosis, and insulin resistance, according to Zhang et al. (2012).

Metabolomics by means of mass spectrometry has recognized that T2DM patients have augmented plasma levels of oxidative stress markers comprising lipid peroxides and advanced glycation end‐products (AGEs) (Lima et al. 2022). These biomarkers assist us in understanding how the disease is affecting metabolic function. Additionally, oxidative damage is further augmented by nutritional variations that foodomics has exposed to occur in T2DM patients, such as reduced glutathione and other endogenous antioxidants (Zhao et al. 2015). These consequences propose that foodomics is useful for the identification of T2DM paths and molecular markers.

## 5 Foodomics in the Identification of Biomarkers for Early Diagnosis and Risk Prediction

The management of T2DM is mainly based on initial diagnosis and risk prediction. Methods taken at the right time can avert or postpone the development of the disease. The usage of foodomics technologies has greatly enhanced our knowledge of potential biomarkers for risk assessment and initial diagnosis. Molecules that designate the occurrence, development, or potential danger of a disease are recognized as biomarkers. In foodomics, some lipids, proteins, and metabolites have been recognized as good biomarkers related to the etiology and development of T2DM.

### 5.1 Metabolomics and Biomarkers

When observing for biomarkers for T2DM, metabolomics is an effective tool. Metabolic plasma or urine profiling of people who are at risk of T2DM may demonstrate unique metabolic markers that may differentiate among prediabetes, normal glucose tolerance, and T2DM, according to systematic reviews of numerous studies. Certain lipid metabolites and variations in amino acid composition, comprising BCAAs, are potential biomarkers for glucose insensitivity and insulin resistance (Vanweert et al. 2022). These biomarkers are capable of recognizing metabolic derangements even when the indications of T2DM or linked problems do not yet exist.

Foodomics recognized several other T2DM‐related metabolites, including lipids and amino acids. T2DM has metabolic profiles with advanced concentrations of glycolytic intermediates and inferior concentrations of certain energy‐related metabolites, e.g., acetylcarnitine, as resolved by Zhang et al. (2025). These are the changes representative of how the metabolism adapts to insulin resistance and glucose derangements. Alterations in gut microbiota conformation and its resultant metabolites may subsidize the development and progression of T2DM Intestinal microbiota‐derived metabolites like short‐chain fatty acids (SCFAs) could control insulin sensitivity and inflammation; the metabolites can be profiled by means of foodomics policies (Hosomi et al. 2022). These consequences form the foundation of monitoring the conformation and activity of the gut microbiota, an integral constituent of a combined risk prediction method for T2DM.

There have been numerous case studies that have been capable of indicating that foodomics can uncover metabolic variations and detect biomarkers that can be used to predict T2DM. For instance, Zhang et al. (2021) studied the outcome of a polyphenol‐rich diet on the metabolism of T2DM. The insulin sensitivity and oxidative stress indicators were meaningfully reduced in the study. This case study designates the use of foodomics in assessing the effects of dietary interference on T2DM development and on the determination of the metabolic alterations associated with it.

Albu et al. (2010) showed a very significant work that studied the metabolic effects of a one‐year diet and exercise intervention in T2DM patients. Metabolic profiles of the patients changed significantly after the intervention, as designated by metabolomic examination. This was followed by augmented insulin sensitivity, decreased obesity, and modifications in lipid and amino acid metabolism. Finding biomarkers that mirror the efficiency of lifestyle intervention in controlling T2DM is a primary aim of foodomics, as established here. In summary, foodomics delivers a robust platform for learning more about the metabolic diseases that cause T2DM. Foodomics assists us in better escalating biochemical disturbances such as insulin resistance, oxidative stress, and abnormal glucose metabolism that are at the core of T2DM by giving a whole picture of lipids, proteins, and metabolites. In conclusion, foodomics has vast potential to disclose early biomarkers that may lead to better risk stratification, earlier diagnosis, and more specific interventions to postpone or prevent disease development. Individualized prevention and treatment of T2DM must be led by follow‐up studies authorizing these markers and retaining in the limelight the role of gut flora and diet.

## 6 Role of the Gut Microbiome in T2DM Development and Foodomics‐Based Interventions

A refined array of microbes existing within the human intestines, or gut microbiome, affects a host of determinants of health, fluctuating from digestion and metabolism to immune function and establishing diseases like T2DM. There is increasing evidence pointing to the fact that variations in gut microbiota are mainly the cause of metabolic aberrations associated with T2DM. With a firm foundation upon which to make T2DM therapies that target the microbiome, foodomics, an interdisciplinary investigation platform that puts on omics technology to inspect the interface between the food and biological systems, is the solution.

### 6.1 Gut Microbiome and T2DM

The structure and function of the gut microbiome influence the onset and progression of T2DM, according to strong evidence. Dysbiosis, a state of disturbance in the gut microbial community, has also been attributed to three key features of T2DM: insulin resistance, systemic inflammation, and generalized metabolic dysfunction (Borgundvaag et al. 2021).

Short‐chain fatty acids like butyrate, acetate, and propionate, which are produced by gut microbiota metabolism of dietary substrates (Henneke et al. 2022), are also of central importance in enhancing insulin sensitivity, regulating lipid metabolism, and regulating inflammation. Where dysbiosis is involved, T2DM patients have reduced numbers of beneficial bacteria implicated in the production of SCFAs, such as *Bacteroidetes* and *Firmicutes*, and increased numbers of pathogenic bacteria, such as *Proteobacteria* and *Firmicutes*, which under other circumstances can be pathogenic. Intestinal permeability and endotoxemia are instigated by an imbalance of the microbes, thereby inducing inflammation within the body. Inflammation is one of the key causes of insulin resistance and impairment of glucose homeostasis, as specified by Zhao et al. (2015).

The gut microbiota also influences T2DM through the gut‐brain‐liver axis. Hepatic metabolism may be changed, and pro‐inflammatory cytokines may be generated when dysbiosis interferes with neuroendocrine signaling within the gut. T2DM formation consequences from a complex series of coupled mechanisms leading to inflammation, insulin resistance, and metabolic dysregulation, Shen et al. (2023) stated.

#### 6.1.1 Influence of Diet on the Microbiome in Diabetic Patients

A person's diet plays an important role in altering the gut microbiome of an individual. The composition and metabolic activity of a T2DM patient can be manipulated by making dietary changes (Su et al. 2023). Systemic inflammation can be reduced and insulin sensitivity optimized by inducing beneficial microbial communities through fiber‐rich, prebiotic, and polyphenol‐containing diets (Zhang et al. 2023).

Conversely, microbiome dysfunctions increase insulin resistance in individuals whose diets are dominated by high‐refined carbohydrates and unwanted fats. Type 2 diabetic gut microbiome has also reported promising outcomes from interventions such as intermittent fasting (IF). Conforming to a systematic review and meta‐analysis of T2DM trials, IF not only enhances metabolic parameters such as fasting glucose and insulin sensitivity but also alters the composition of the gut microbiota favorably (Borgundvaag et al. 2021).

It has been established through research that IF decreases harmful *Firmicutes* and enhances beneficial *Bacteroidetes* and other bacterial groups. T2DM patients have gut microbial communities that are progressively healthier when on a high‐fiber diet consisting of fruits, vegetables, and whole grains. SCFA‐producing microbes are stimulated by prebiotic fiber to proliferate. Type 2 diabetic patients who ate higher amounts of fiber had a healthier gut microbial community, lower levels of inflammation markers, and higher insulin sensitivity, according to a study by Henneke et al. (2022).

#### 6.1.2 Foodomics and Its Role in Gut Microbiome‐Based Interventions for T2DM

One of the best methods for realizing the influence of nutrition on human health and gut microbiome is foodomics, which integrates genomes, proteomics, metabolomics, and microbiomics. Scientists can better understand the activities of food components on metabolic disorders such as T2DM by analyzing their interactions with microbial communities (Singh et al. 2024). For instance, foodomics investigation has ascertained that the high‐polyphenol Mediterranean diet contributes to the promotion of friendly bacteria such as *Bifidobacterium* and *Lactobacillus*. Both microbial species are promoted in maintaining the strengthening of the intestinal barrier and inhibiting systemic inflammation (Zheng and Chen 2014).

To give a complete image of how nutritional consumption affects metabolic health, foodomics technology can also monitor the metabolic derivatives of the gut microbiota following exposure to the individual dietary constituents. Individualized dietetic advice is most likely the most useful application of foodomics in T2DM. Foodomics can support individualized dietetic interventions with greater potential for therapy through the integration of information regarding diet consumption and individual microbiota patterns. For instance, Keijer et al. (2024) established that metabolic and glycemic control in type 2 diabetes patients was improved by personal microbiome topology individualized dietetic treatments. Finally, the gut microbiome influences insulin sensitivity, inflammation, and metabolic control in a very significant way in T2DM etiology. One of the most potent effects on the microbiome is the dietary component, and foodomics gives us a new vision of the complex interactions between food and microbiota. Personalized prevention and treatment of T2DM are directed by foodomics via microbiome‐targeted dietary interventions (Table 2). Table 2 depicts the case studies highlighting metabolic and gut microbiome insights in T2DM through foodomics approaches.

**TABLE 2:** Case studies highlighting metabolic and gut microbiome insights in T2DM through foodomics approaches.

Category	Study design	Key techniques	Major findings	Pathways/Genes identified	References
Metabolic dysregulation in T2D	Plasma metabolomics in T2DM patients versus controls	NMR spectroscopy, LC–MS/MS	Elevated branched‐chain amino acids (BCAAs: leucine, isoleucine, valine) correlated with insulin resistance	mTOR/S6K1 signaling pathway activation; *BCKDHA* gene dysregulation	Wassink et al. (2007), Chen et al. (2020)
	NMR‐based metabolic profiling of T2DM progression	^1^H‐NMR, multivariate analysis (PCA/PLS‐DA)	Lipid shifts (↑ceramides, ↓lysophosphatidylcholines) and amino acid imbalances (↑phenylalanine)	Sphingolipid metabolism; *FADS1/2* polymorphisms linked to lipid alterations	Yen et al. (2023), Hasin et al. (2017)
	1‐year diet/exercise intervention in T2DM	GC–MS metabolomics, insulin clamp assays	Improved glucose disposal (+28%) and ↓hepatic fat; ↑butyrate‐producing gut microbes	*PPARγ* activation; *SCD1* downregulation in adipose tissue	Albu et al. (2010), Backman et al. (2019)
	Serum metabolomics for early T2DM detection	UHPLC‐QTOF‐MS, machine learning	5‐metabolite panel (↑α‐hydroxybutyrate, ↓linoleic acid) predicted prediabetes (AUC = 0.91)	Glutathione metabolism; *GCKR* rs1260326 variant association	Zhang et al. (2025), Fitipaldi et al. (2018)
	Selenium supplementation in T2DM	ICP‐MS, redox proteomics	Selenium reduced oxidative stress (↓MDA, ↑GPx activity) and improved HOMA‐IR	*SELENOP* expression linked to insulin sensitivity; Nrf2/ARE pathway modulation	Steinbrenner et al. (2022), Chevalier and Fénichel (2015)
Gut microbiome in T2D	*Blautia wexlerae* supplementation in obese T2DM mice	16S rRNA sequencing, metagenomics, LC–MS metabolomics	↑*Blautia* abundance (↓Firmicutes/Bacteroidetes ratio) improved glucose tolerance (+35%)	Butyrate synthesis (*butyryl‐CoA transferase* genes); GLP‐1 secretion modulation	Hosomi et al. (2022), He et al. (2021)
	Multi‐omics analysis of gut microbiota‐diet interactions	Shotgun metagenomics, fecal SCFA profiling	*Parasutterella*‐driven fatty acid biosynthesis (↑palmitoleic acid) linked to insulin resistance	*ACC1* and *FASN* upregulation; PPARα pathway inhibition	Henneke et al. (2022), Armenteros et al. (2024)
	Polyphenol‐rich diet intervention in T2DM	UPLC‐TQ‐MS, 16S rRNA sequencing	↑ *Akkermansia muciniphila* (+4.2‐fold) and ↓HbA1c (−1.2%); polyphenol metabolites (urolithin A) detected	Aryl hydrocarbon receptor (AhR) activation; *CLDN1* tight junction enhancement	Zhang et al. (2021), Braconi et al. (2018)
	Prebiotic (inulin) trial in T2DM patients	Metatranscriptomics, NMR metabolomics	↑*Bifidobacterium* (↓*Escherichia*); ↓LPS (−18%) and ↑butyrate (+2.5‐fold)	*TLR4/NF‐κB* pathway suppression; *FFAR2* (SCFA receptor) upregulation	Song et al. (2022), Ďásková et al. (2023)
	Meta‐analysis of gut microbiome in T2DM	16S rRNA/whole‐genome sequencing (25 studies)	Consistent depletion of *Roseburia* and ↑ *Ruminococcus gnavus* in T2DM (FDR < 0.05)	Bacterial butyrate synthesis genes (*butK*, *butD*) inversely correlated with HOMA‐IR	VanEvery et al. (2023), Eddy et al. (2020)
Diet–microbiome interactions	High‐fiber diet (30 g/day) in T2DM	GC‐FID (SCFA analysis), ITS sequencing	↑Acetate (+40%) and propionate (+25%); improved HOMA‐IR (−22%)	*PEPCK* and *G6PC* hepatic gluconeogenesis gene downregulation	Mussap et al. (2021), Guo et al. (2022)
	Mediterranean diet (MedDiet) RCT in T2DM	Metagenomics, LC–MS lipidomics	↑Microbial α‐diversity (+15%), ↓oxLDL (−12%) and ↑omega‐3 PUFA levels	*Bacteroides*‐driven bile acid metabolism (↑FXR signaling)	Kautzky‐Willer et al. (2023), Bordoni and Capozzi (2014)
	Ketogenic diet (KD) in obese T2DM patients	16S rRNA sequencing, serum β‐hydroxybutyrate assays	KD shifted microbiota (↑*Prevotella*, ↓*Bifidobacterium*); improved HbA1c (−1.8%)	*HMGCS2* ketogenesis gene upregulation; *GLUT4* translocation enhancement	Pellegrini et al. (2024), Borgundvaag et al. (2021)
	Whole‐food (barley β‐glucan) intervention	HPLC‐MS, metaproteomics	↑*Christensenellaceae*; ↓postprandial glucose (−20%) via delayed starch digestion	*AMY1A* (amylase) inhibition; *SGLT1* downregulation in enterocytes	López‐Moreno et al. (2021), Idehen et al. (2020)
	Prebiotic (FOS/GOS) efficacy evaluation	NMR metabolomics, qPCR (microbial taxa)	FOS ↑*Lactobacillus* (+3.1‐fold); GOS ↓*Desulfovibrio* (↓H2S production)	Microbial *galE* (galactose metabolism) gene enrichment; ↓TMAO synthesis	That et al. (2022), Chung et al. (2018)

## 7 Mechanisms of Foodomics‐Based Microbiome Interventions in T2DM

Foodomics‐driven microbiome therapeutics address common major pathways in T2DM. To begin with, by supporting the generation of favorable metabolites like SCFAs, gut microbiota diversity can be utilized to facilitate less inflammation and enhanced insulin sensitivity. SCFAs are generated as a result of the fermentation of dietary fiber by gut bacteria (Faulkner et al. 2020). Improvements in insulin sensitivity and reinforcement towards synthesizing hormones like GLP‐1 that increase glucose homeostasis have been reported to regulate glucose metabolism (Vanweert et al. 2022). Second, the presence of ingredients in foods that directly manipulate gut flora is searched for via foodomics. Even a study indicates that polyphenols serve as prebiotics, supporting the growth of beneficial gut flora by preventing pathogenic bacteria growth (Zhang et al. 2021).

HMG has been established through such foodomics insights with the development of specific therapies to enhance gut health in diabetic individuals. Foodomics enables the identification of possible biomarkers of how an individual would react to a specific diet, permitting guidance towards the optimal customization of therapy. For instance, T2DM subjects' metabolomic profiling might provide insight into markers at the beginning of insulin resistance (Sébédio 2017).

The gut microbiota plays a huge role in T2DM through inflammation and insulin resistance levels that create metabolic disorders. Nutrition is critical to the microbiome and the ability of different dietary styles –like those rich in fiber and polyphenols –to increase microbiota diversity in enhancing metabolic fitness (Ghallab et al. 2024). Foodomics enables personalized nutrition because it is one of the functional tools for making sense of such a complex situation between food and the microbiota and T2DM. Foodomics may support the upscaling of microbiome‐derived therapeutics for predicting and treating T2DM by identifying biomarkers of microbial reactions and food bioactive constituents.

### 7.1 Precision Nutrition for T2DM Management

Using foodomics, precision nutrition for controlling T2DM tailors dietary recommendations based on each individual's unique genetic, metabolic, and microbiome evidence. Individualized nutrition recommendations can be produced by looking at omics profiles, such as genomes, metabolomics, and microbiomics to achieve supreme glycemic control and minimize the danger of impediments. This method helps to moderate the amount of intake based on individual differences in metabolic demands, increases insulin sensitivity, and may have helpful effects on long‐term consequences, such as decreasing the chances of renal failure and cardiovascular diseases commonly related to diabetes.

### 7.2 Personalized Diet Recommendations Based on Omics Data for T2DM Management

Personalized diet programs have a firm basis due to the rapid evolution of omics technologies such as foodomics, genomics, and metabolomics. Foodomics improves glycemic management and alleviates issues related to T2DM therapy through the utilization of genetic and metabolic profiling to recommend personalized diets. The application of these omics techniques in personalized nutrition allows physicians to understand the individualized metabolic response of each patient (Li et al. 2019). Such personalized and effective treatments are therefore accorded to those with T2DM. In discussing the importance of individually tailored diet suggestions based on omics data for administering T2DM, the present study highlights probable foodomics benefits: better health results in greater glycemic control, fewer complications, and inclusive well‐being.

#### 7.2.1 Omics Data for Tailored Nutritional Interventions

Since omics technologies, be it genomics, metabolomics, or even foodomics because they can unravel the individual differences in a distinct genetic, metabolic, and even microbiome profiling, would most likely gain more substantial recognition regarding their capacity for precision medicine, it makes sense to view these data based on developing individual nutrition plans customized toward each individual (Alba et al. 2019; Wang and Hu 2018).

The primary goal of the omics area of foodomics is the study of food's bioactive components and how they affect human health (Damarov et al. 2024). This equipment allows healthcare providers to scrutinize how numerous meals distress an individual's metabolic procedures based on their genetic conformation and medical history, thereby creating dietary programs that exploit glucose metabolism and enhance insulin sensitivity (Merino 2022). For instance, genetic markers can determine how a person's body processes proteins, lipids, and carbohydrates, thereby manipulating some dietary variations to reduce the spikes in blood glucose (Gloyn and Drucker 2018). This is advanced by foodomics, which determines bioactive compounds in food, such as fiber or polyphenols, that can increase insulin sensitivity or regulate blood sugar levels (Yang et al. 2021). This recovers the overall effectiveness of dietary advice by permitting nutritional interventions that are precisely targeted to the metabolic needs of individuals with T2DM (Table 3). Table 3 depicts the precision nutrition for T2DM management.

**TABLE 3:** Precision nutrition for T2DM management.

Nutrient/Intervention	Dosage recommendation	Personalization basis	Impact on glycemic control	References
Omega‐3 fatty acids	1–2 g/day	Genetic predisposition to inflammation	Reduces fasting glucose and triglycerides	Wang and Hu (2018)
Probiotic supplementation	≥ 10 billion CFU/day	Gut microbiota composition	Improves HbA1c and insulin sensitivity	Ben‐Yacov and Rein (2022)
Fiber‐rich diet	25–30 g/day	Metabolic profiling	Enhances postprandial glucose response	de Toro‐Martín et al. (2017)
Low‐glycemic‐index foods	Glycemic load < 15/meal	SNPs in glucose transporter genes	Stabilizes blood sugar fluctuations	Shamanna et al. (2020)
Barley β‐glucans	3 g/day	Dietary metabolomic data	Improves insulin sensitivity	Idehen et al. (2020)
Polyphenol‐rich foods	≥ 200 mg/day	Genotype and gut microbiome compatibility	Reduces oxidative stress and inflammation	Singh et al. (2024)
Personalized meal timing	Individualized	Circadian rhythm and metabolic rate	Optimizes glucose metabolism	Alaofè et al. (2024)
Vitamin D supplementation	1000–4000 IU/day	Genomic vitamin D receptor profiling	Enhances insulin secretion	Robertson et al. (2024)
Protein‐enriched diet	1.2–1.6 g/kg body weight	Genetic variation in protein metabolism	Improves satiety and glycemic control	Garg and Heber (2024)
Plant‐based diet	> 60% plant‐based foods	Nutrigenomics and microbiota diversity	Reduces HbA1c and weight	Voruganti (2023)
Functional oat products	≥ 50 g/day	SNPs affecting lipid metabolism	Lowers LDL‐C and improves glucose tolerance	Janda et al. (2019)
Chromium picolinate	200–400 μg/day	Insulin resistance phenotypes	Enhances insulin action	Ramos‐Lopez (2024)
Mediterranean diet	Tailored to SNPs	SNPs in fat metabolism genes	Reduces fasting glucose and HbA1c levels	Tuncay and Ergoren (2020)
Whole grain cereal intake	2–3 servings/day	Fiber and resistant starch profiles	Lowers postprandial glucose levels	Wu et al. (2020)
Flaxseed supplementation	30 g/day	Omega‐3 metabolism and gut health	Lowers HbA1c and reduces inflammation	Mortazavi and Gutierrez‐Osuna (2023)
Functional soy products	≥ 50 g/day	SNPs in isoflavone metabolism	Reduces fasting blood glucose	Merino (2022)
Digital nutrition monitoring	App‐based	Personalized digital twin modeling	Reduces HbA1c with real‐time feedback	Shamanna et al. (2021)
Antioxidant‐rich foods	Tailored intake	Metabolomic profiling	Reduces oxidative damage to β‐cells	Fernández‐Ochoa et al. (2021)
Ketogenic diet	≤ 50 g carbs/day	Metabolotyping for fat utilization	Enhances insulin sensitivity	Pigsborg and Magkos (2022)
Personalized hydration plan	2–3 L/day	Urinary metabolomics	Optimizes glucose excretion	LeVatte et al. (2022)

### 7.3 Potential Benefits in Improving Glycemic Control and Preventing Complications

There is considerable scope for using personalized nutrition tailored to omics data to increase glycemic control and reduce risks associated with T2DM complications. Perhaps the biggest issue with controlling T2DM over time is that it is unable to maintain normal blood sugar levels. It then addresses the concern by individualizing dietary advice that suits a client's unique reaction to food in precision nutrition while also reducing levels of HbA1c, which forms a vital point of assessment concerning long‐term glucose control (Antwi 2023).

Precision dieting has been noted to affect lipid, insulin, and glucose regulation improvements (Shamanna et al. 2020). In addition, among patients with T2DM, tailored nutritional interventions lower the rates of all causes of outcomes like neuropathy, renal disease, and heart disease (Garg and Heber 2024). These tailored treatments, through enhanced nutrition absorption and regulation of underlying metabolic imbalances, help to decrease oxidative stress and inflammation, two primary causes of issues in diabetic patients (Ben‐Yacov and Rein 2022). Moreover, foodomics‐based recommendations may identify specific foods and nutrients that can fulfill each individual's unique health needs. This may lead to improved glucose control and a better quality of life (Alaofè et al. 2024).

### 7.4 The Role of Digital Innovations in Precision Nutrition

Precision nutrition and digital innovation have increased the potential of personalized dietary recommendations for T2DM treatment (Alba et al. 2019). Continuous following of blood sugar levels, eating patterns, and other health pointers is allowable by big data and AI‐powered digital platforms (Mortazavi and Gutierrez‐Osuna 2023). For example, digital twin technology can create a cybernetic model of a person's metabolic progressions and, with the ability to simulate many dietary interventions' effects, alter, in real time, a personalized nutrition plan (Shamanna et al. 2021). When used with omics data, these technologies have the unprecedented opportunity to manage T2DM to an unknown accuracy (Kupai et al. 2022).

Real‐time, dynamic personalized nutrition advice is created using AI and machine learning algorithms, improving individualized nutrition by predicting how specific foods affect an individual's glycemic reaction (Robertson et al. 2024). Integrating these advanced technologies further enhances the effectiveness of dietary treatments and facilitates better adherence through simple, tailored nutrition advice. Augmented by omics technologies, precision nutrition might be a revolutionary strategy to avert and treat T2DM. Improving glycemic management, risks of problems, and overall health consequences could be contingent on personalized recommendations based on exceptional genetic, metabolic, and microbiome data. The more the methodology evolves, the better the integration of omics data into clinical training, which would also deliver even more accuracy in tailoring nutrition therapies for patients with T2DM. Additionally, through the integration of digital technology and AI, real‐time, actionable visions into dietary management advance the probability of precision nutrition and make it a promising tool for controlling chronic illnesses such as T2DM (Merino 2022; Wang and Hu 2018).

### 7.5 Foodomics and Functional Foods in Diabetes

**FIGURE 3:**
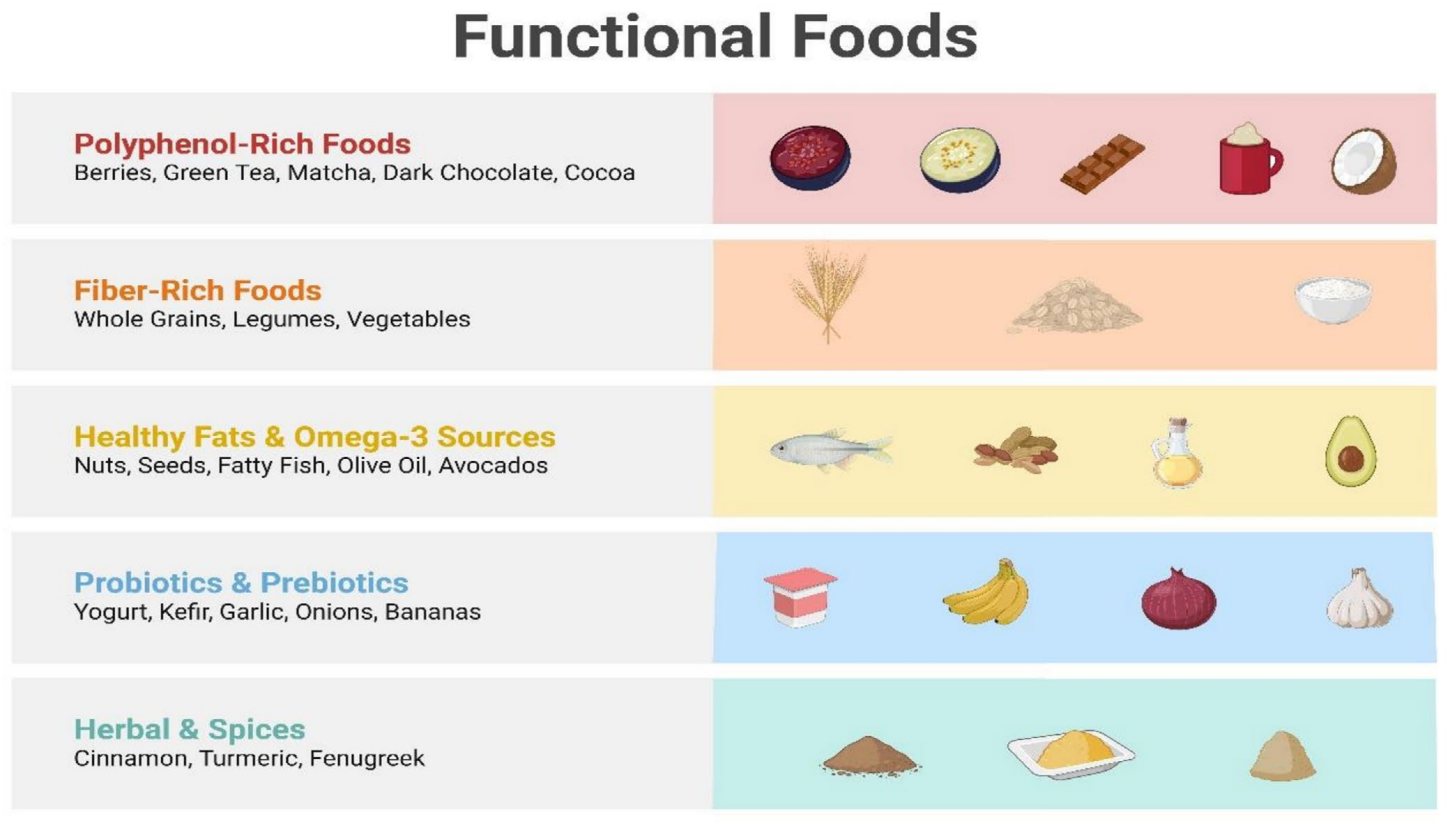
Functional food products.

Because of its rising prevalence and the risk of complications such as cardiovascular disease, neuropathy, and retinopathy, diabetes, particularly T2DM, has become a worldwide health issue. Dietary modifications and other lifestyle interventions are commonly used to manage this disease. Foodomics, the study of diet and its bioactive compounds using omics technologies, including transcriptomics, proteomics, metabolomics, and genomics, delivers promising new perceptions on the conceivable role of food in the anticipation and management of diabetes. Interest in functional foods, that is, foods improved with bioactive composites that are more than basic nutrition, in diabetes management has also been rising. This report evaluates the role that foodomics plays in classifying bioactive compounds that enhance the therapy of T2DM and also assesses functional foods like barley, oats, and others related to the power to control diabetes (Figure 3). Figure 3 shows the functional food products.

## 8 Foodomics in Diabetes Research

Sophisticated analytical techniques in foodomics technology also give profound insights into multifaceted relationships between food ingredients and human health (Abdurahman et al. 2019). It further found how various dietary fibers and polyphenols affect the progression of diabetes by altering specific pathways of biological processes (Yang et al. 2021). Better insights into how numerous dietary components may influence insulin sensitivity, inflammation, and metabolism, all factors crucial in the management of T2DM, may be gained by the application of omics technology in mapping and characterizing the molecular conformation of food (Yang et al. 2016).

Instead, this has considered the potential medicinal bioactive compounds, particularly polyphenol compounds, in plant‐based food diets. Possible options among them are anti‐inflammatory, insulin‐sensitive, and antioxidants like flavonoids and phenolic acids. According to this article, such substances may interfere with cell signaling pathways, which may cause variations in human glucose metabolism. For instance, Si et al. (2021) have proved that polyphenols in fruits, vegetables, and whole grains improve insulin sensitivity and reduce new cases of T2DM while lowering blood sugar levels. Thus, foodomics has made it possible to know the exact food ingredients for these health benefits. Such foodomics research includes classifying the molecular pathways through which the bioactive compounds exert their actions. By applying techniques such as high‐resolution mass spectrometry, it is possible to analyze complex food matrices and determine which bioactive compounds induce health‐enhancing effects. In this regard, such a technique will provide an improved understanding of the mechanisms underlying how dietary habits and food factors contribute to the onset and progression of chronic diseases like diabetes (Yang et al. 2021; Ortea 2022).

### 8.1 The Role of Functional Foods in Diabetes Management

**FIGURE 4:**
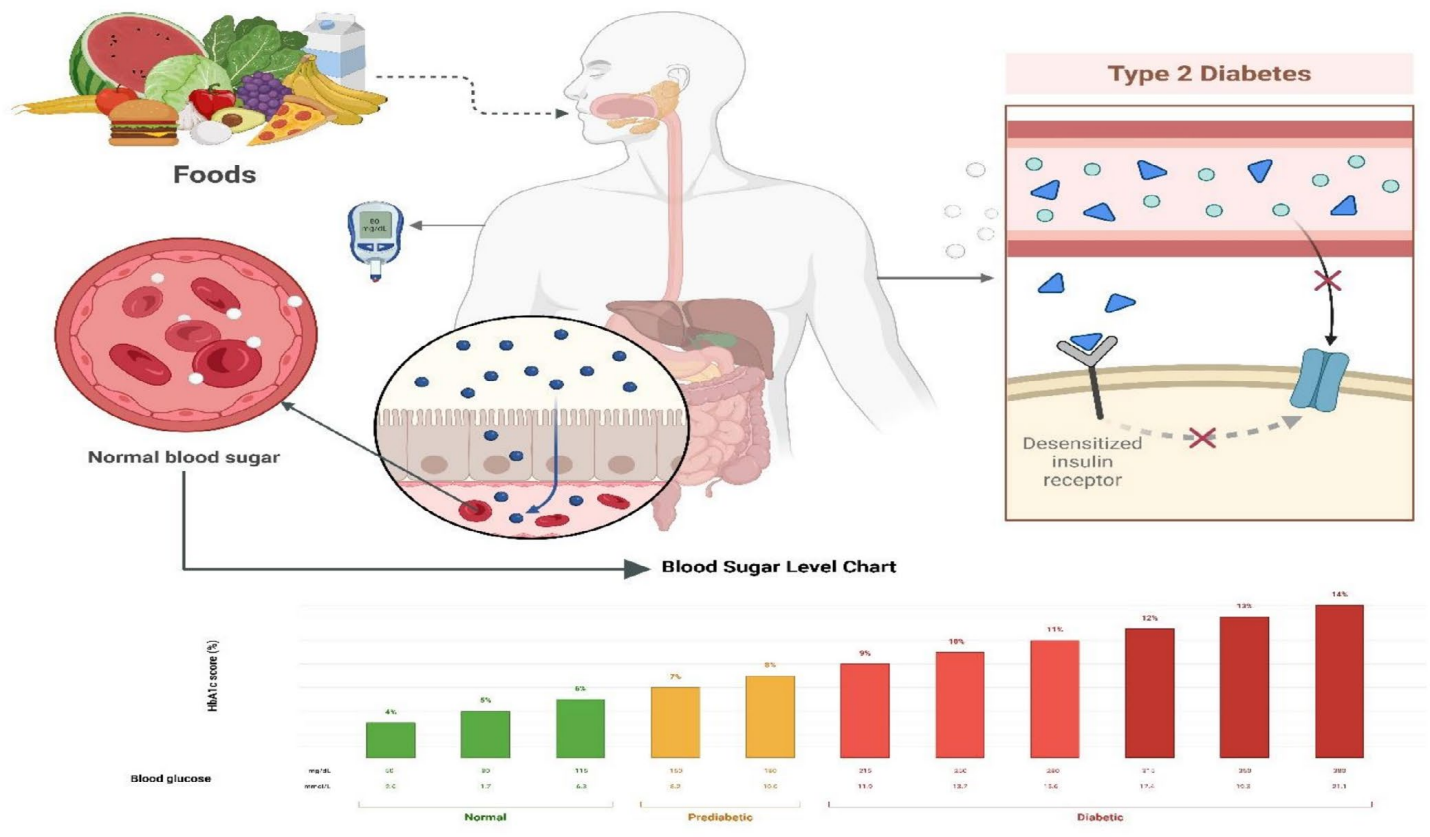
Functional foods and their impact on blood glucose regulation in T2DM.

Functional foods, as operationalized by Ren et al. (2019), not only act as a source of nutrients but they also have health benefits. Functional foods tend to carry high amounts of bioactive compounds like fiber, polyphenols, and good fats, which have been related to improved metabolic health (Guo et al. 2022). Functional foods can play a role in modifying T2DM by the respite of inflammation, improving blood glucose regulation, and improving insulin sensitivity (Braconi et al. 2018). Based on the several prospective benefits of using cereals such as barley and oats to cope with diabetes, these are among the most beneficial foods (Figure 4). Figure 4 shows the functional foods and their impact on blood glucose regulation in T2DM.

#### 8.1.1 Barley: A Functional Food for Diabetes

Barley is a cereal grain that has been shown to have massive effects on glucose metabolism and insulin sensitivity due to its elevated concentration of soluble fiber, predominantly β‐glucans (Armenteros et al. 2024). Bioactive β‐glucans, which are recognized to decrease postprandial glucose peaks and improve insulin sensitivity, have also been established as specified in a study by Geng et al. (2022). According to the majority of investigations, barley consumption varies lipid and blood glucose levels, which can reduce T2DM (Idehen et al. 2020). In addition to being an antidiabetic drug, barley comprises polyphenols with anti‐inflammatory and antioxidant properties, such as flavonoids and phenolic acids (Shvachko et al. 2021).

#### 8.1.2 Oats: Another Beneficial Grain

Oat is also a cereal grain that has gained importance due to its application in diabetes management (Agrawal et al. 2013). The foodstuffs are elevated in soluble fiber, such as β‐glucan, which can advance glycemic control and lower glucose absorption (Ellett et al. 2013). Clinical trials have shown that oat intake improves insulin sensitivity and decreases fasting blood glucose levels in T2DM patients (Janda et al. 2019). Besides, a significant amount of polyphenols, known to inhibit inflammation and serve as antioxidants, in oats could potentially mitigate complications resulting from diabetes (Sikand et al. 2015).

Oats contain phenolic complexes that comprise the anti‐inflammatory and antioxidant qualities of avenanthramides in the accumulation of fiber and polyphenols. Such chemicals are known to have effects of improving vascular health as well as the level of insulin resistance (Giacco et al. 2013). These make oats valuable as a functional food in diabetes cases due to the occurrence of its ingredients.

#### 8.1.3 Other Grains and Functional Foods for Diabetes

Bioactive compounds from wheat, rye, and quinoa have been shown to assist in managing diabetes, besides those from barley and oats. Rye has high fiber and different phenolic acid profiles, and it was reported that they increased insulin sensitivity and glycemic management (Sidhu et al. 2007). Frequently termed a “supergrain,” quinoa is rich in vitamins, minerals, and bioactive substances, such as polyphenols and saponins. The latter are compounds that provide anti‐inflammatory and antioxidant properties, assisting in diabetes (Brennan and Cleary 2005).

In addition, there is also interest in understanding whether the regulated sprouting of germinated grains improved their bioactive chemical profiles. Nelson et al. (2013) contend that sprouted cereals usually have elevated antioxidants, fiber, and phenolic compounds that promote better metabolic health and avert and potentially treat diabetes. Advances in sophisticated analysis tools used in foodomics science give an apt perspective on personal bioactive constituents in food, their role in metabolic health, and how they aid in managing diabetes (Jiang et al. 2020).

Foodomics' work with polyphenols, fibers, and other constituents offers increased benefits in controlling insulin sensitivity, reducing blood sugar, and eliminating inflammation that becomes major in T2DM. These bioactive compounds abound in functional foods such as barley, oats, and other cereals and contain a lot of potential to be incorporated into programs for diabetes control. With advancing science, the identification and verification of functional foods for diabetes will become an increasingly important aspect of foodomics. This will disclose new prospects in individualized nutrition and the enterprise of effective interventions to help type 2 diabetics or those at risk of developing diabetes (Table 4). Table 4 depicts the functional foods and bioactive compounds in diabetes management.

**TABLE 4:** Functional foods and bioactive compounds in diabetes management.

Bioactive compound/Functional food	Source	Mechanism of action	Omics technology	Diabetes‐related benefit	References
Polyphenols	Green tea, berries	Antioxidant, anti‐inflammatory	Foodomics	Improves insulin sensitivity	Yang et al. (2021), Si et al. (2021)
β‐Glucans	Barley, oats	Slows glucose absorption	Metabolomics	Reduces postprandial glucose levels	Geng et al. (2022), Brennan and Cleary (2005)
Resistant starch	Bananas, legumes	Enhances gut microbiota	Metagenomics	Reduces insulin resistance	Balkir et al. (2021)
Anthocyanins	Purple corn, berries	Reduces oxidative stress	Proteomics	Improves β‐cell function	Si et al. (2021)
Ferulic acid	Whole grains	Modulates glucose metabolism	Transcriptomics	Lowers fasting glucose levels	Fernández‐Ochoa et al. (2021)
Soluble fiber	Oats, psyllium	Increases satiety, reduces GI transit	Nutrigenomics	Lowers HbA1c levels	Janda et al. (2019)
Lignans	Flaxseeds, sesame	Phytoestrogenic, antioxidant	Foodomics	Reduces insulin resistance	Idehen et al. (2020)
Proanthocyanidins	Cocoa, apples	Anti‐inflammatory	Metabolomics	Lowers blood glucose levels	Zhang et al. (2021)
Isoflavones	Soy products	Estrogen receptor modulation	Transcriptomics	Improves insulin sensitivity	Braconi et al. (2018)
Magnesium	Whole grains, nuts	Cofactor in glucose metabolism	Elementomics	Lowers risk of type 2 diabetes	Singh et al. (2024)
Tocopherols	Nuts, seeds	Lipid peroxidation inhibition	Lipidomics	Improves lipid profile	Vimaleswaran et al. (2015)
Omega‐3 fatty acids	Fish, flaxseed oil	Anti‐inflammatory	Nutrigenomics	Reduces inflammatory markers	Srour et al. (2020)
Phytosterols	Vegetable oils	Cholesterol‐lowering effect	Lipidomics	Reduces cardiovascular risk in diabetes	Lorber (2014)
Catechins	Green tea	Improves insulin signaling	Proteomics	Enhances glucose uptake	Yang et al. (2021)
Carotenoids	Carrots, spinach	Antioxidant	Metabolomics	Lowers oxidative stress	Balkir et al. (2021)
Curcumin	Turmeric	NF‐κB inhibition	Transcriptomics	Reduces inflammation	Si et al. (2021)
Pectins	Apples, citrus	Slows gastric emptying	Metabolomics	Lowers postprandial glucose levels	Shvachko et al. (2021)
Plant sterols	Fortified foods	Reduces LDL cholesterol	Lipidomics	Protects cardiovascular health in diabetes	Nelson et al. (2013)
Chlorogenic acid	Coffee, green tea	Modulates glucose transporters	Foodomics	Improves glucose tolerance	Zhang et al. (2021)
Saponins	Legumes, quinoa	Anti‐inflammatory	Proteomics	Enhances insulin signaling	Ren and Li (2019)
Quercetin	Onions, apples	Inhibits α‐glucosidase	Foodomics	Reduces postprandial glucose spike	Cheng et al. (2024), Si et al. (2021)
Zeaxanthin	Corn, egg yolk	Retinal protection	Metabolomics	Reduces the risk of diabetic retinopathy	Ortea (2022)
Phytic acid	Whole grains	Mineral chelation	Transcriptomics	Lowers risk of cardiovascular complications	Singh et al. (2024)
Alkylresorcinols	Rye, wheat bran	Antioxidant	Nutrigenomics	Reduces oxidative stress	Balkir et al. (2021)
Gamma‐oryzanol	Rice bran	Modulates lipid metabolism	Metabolomics	Lowers cholesterol levels	Santos et al. (2022)
Dietary nitrates	Beets, leafy greens	Improves nitric oxide availability	Metabolomics	Lowers blood pressure	Misra and Misra (2020)

## 9 Food Safety and Risk Assessment in T2DM

### 9.1 Emerging Contaminants and Their Impact on Diabetes: The Role of Foodomics

Food safety issues are increasingly being tied to the growth in T2DM prevalence universally: exposure to novel toxins (Chung et al. 2018). The integration of genomics, proteomics, and metabolomics foodomics will lead to the new development of identifying diverse types of toxins, such as pesticides, heavy metals, and EDCs, which increase or otherwise alter the risk for T2DM (Drincic et al. 2017).

#### 9.1.1 Pesticides and Diabetes Risk

Besides being heavy environmental pollutants applied in agriculture, pesticides have also been associated with metabolic disorders (Castro‐Muñoz et al. 2022). A study shows that long‐term pesticide exposure to humans by occupational and dietary routes containing organochlorine and organophosphate may alter insulin secretion and sensitivity, which causes T2DM (Tyagi et al. 2021).

He et al. (2024) assert that these chemicals bioaccumulate in human tissues and lead to mitochondrial dysfunction and oxidative stress, two main mechanisms involved in the development of diabetes (Chatterjee et al. 2017). Some pesticide metabolites have been detected in blood and urine using foodomics approaches, and the exposure has been related to the induction of diabetes (Nguyen 2023). Agricultural workers are highly vulnerable group due to direct exposure to pesticide residues through food consumption and direct exposure in the field (Ruiz et al. 2018).

#### 9.1.2 Heavy Metals as Diabetogens

Heavy metals, including arsenic, cadmium, lead, and mercury, pollute food and water (Fu et al. 2024). As per Mudumbi et al. (2018), these metals also play a role in the pathophysiology of T2DM by instigating systemic inflammation and compromising the pancreatic beta‐cell activity. The meta‐analysis of Nguyen in 2023 revealed how several heavy metals together increase insulin resistance. For example, arsenic affects the signaling pathways of insulin, changing glucose metabolism; cadmium is related to oxidative stress and chronic hyperglycemia (Liu et al. 2023). Methods of foodomics for heavy metal contamination in food matrices allow the identification of pathways of acquaintance by diet (Table 5) (Su et al. 2024). Table 5 depicts the contaminants and their impact on T2DM.

**TABLE 5:** Contaminants and their impact on T2DM.

Contaminant	Description	Impact on T2DM	References
Pesticides	Chemicals used to protect crops may persist in food and water	Induce insulin resistance via oxidative stress	Tyagi et al. (2021), Ruiz et al. (2018)
Heavy metals (e.g., Lead)	Toxic metals accumulating in soil and water due to industrial pollution	Disrupt beta‐cell function and increase insulin resistance	Liu et al. (2023), Nguyen (2023)
Heavy metals (e.g., Cadmium)	Found in contaminated water, fertilizers, and industrial waste	Affects glucose metabolism and mitochondrial function	Kuo et al. (2013), Lee et al. (2014)
Endocrine disruptors	Chemicals interfere with hormonal balance, including phthalates and bisphenol A (BPA)	Alter insulin signaling pathways and promote metabolic syndrome	Fénichel and Chevalier (2017), He et al. (2024)
Chlorinated pollutants	Persistent organic pollutants (POPs) like PCBs in industrial processes	Chronic inflammation leading to insulin resistance	Lee et al. (2014), Khalil et al. (2023)
Mycotoxins	Toxic compounds from mold growth in improperly stored grains or nuts	Exacerbate oxidative stress and inflammation	Mudumbi et al. (2018), Fung et al. (2018)
Plasticizers	Found in food packaging and containers, often leach into food and beverages	Associated with obesity and impaired glucose tolerance	Sargis and Simmons (2019), Schulz and Sargis (2021).
Food additives	Artificial sweeteners, colorants, and preservatives used to enhance shelf life and taste	Linked to gut microbiome changes and insulin sensitivity reduction	Velmurugan et al. (2017), Gupta et al. (2020)
Acrylamide	Produced during high‐temperature cooking of starchy foods (e.g., frying, baking)	Associated with beta‐cell dysfunction and glucose intolerance	Fung et al. (2018), Petrakis et al. (2017)
Polycyclic aromatic hydrocarbons (PAHs)	Formed during the grilling or smoking of meats	Linked to mitochondrial dysfunction and oxidative stress	Carter and Blizard (2016), Zhang et al. (2024)
Dioxins	By‐products of industrial combustion processes	Induce chronic inflammation, contributing to diabetes onset	Haverinen et al. (2021), Sargis and Simmons (2019)
Mercury	Present in seafood due to ocean pollution	Interferes with pancreatic beta‐cell activity and glucose metabolism	Nguyen (2023), Mudumbi et al. (2018)
Arsenic	Found in contaminated groundwater and rice products	Impairs insulin secretion and increases diabetes risk	Kuo et al. (2013), Neel and Sargis (2011)
Phthalates	Common in plastics and personal care products, transferred through food contact materials	Disrupts endocrine functions and promotes obesity	Velmurugan et al. (2017), Carter and Blizard (2016)
Perfluorinated compounds (PFCs)	Used in non‐stick cookware and water‐resistant products	Implicated in insulin resistance and lipid metabolism alterations	Khalil et al. (2023), Nguyen (2023)
Brominated flame retardants	Found in household items, migrate to food	Causes mitochondrial dysfunction and disrupts glucose metabolism	Petrakis et al. (2017), Mudumbi et al. (2018)
Nitrosamines	Found in cured meats and processed foods	Increase oxidative stress and damage pancreatic beta cells	Fung et al. (2018), Carter and Blizard (2016)
Pesticide residues	Trace amounts remain on fruits and vegetables after application	Increase oxidative stress, impairing insulin sensitivity	Tyagi et al. (2021), Hectors et al. (2011).
Heavy metals (e.g., Arsenic)	Often present in rice, drinking water, and shellfish	Reduces insulin secretion and affects beta‐cell function	Kuo et al. (2013), Gupta et al. (2020)
Residual antibiotics	Found in animal‐based food products due to overuse in livestock	Linked to gut microbiome changes and metabolic disruptions	Velmurugan et al. (2017), Khalil et al. (2023)

#### 9.1.3 Endocrine Disruptors and Metabolic Dysregulation

Specific samples of EDCs include Bisphenol A (BPA), phthalates, and persistent organic pollutants (POPs). These chemicals mimic or inhibit hormonal systems to damage glucose homeostasis (Cavalli et al. 2021). EDCs have been shown to exacerbate insulin resistance, adipogenesis, and beta‐cell dysfunction (Chevalier and Fénichel 2015). For instance, BPA acts through estrogen receptors to alter insulin secretion and sensitivity, while phthalates adjust adipokine levels, thus encouraging systemic inflammation (Velmurugan et al. 2017).

Advanced foodomics analyses have assisted in making EDC residues present in dietary sources easier to categorize, thereby becoming a source of vital information toward risk assessment (Papalou et al. 2019). The incorporation of foodomics into the study of diabetes reveals the very intricate relationship that exists between the new pollutants and metabolic health (Peng et al. 2025). Therefore, T2D risk management involves detecting and reducing dietary exposure to pesticides, heavy metals, and EDCs. Upcoming studies would be best centered on improving the public's awareness of the dangers to food safety and lowering contaminant exposure through improved agricultural techniques while utilizing omics‐based biomarkers to generate precision nutrition plans.

### 9.2 Food Authenticity and Safety in Diabetes Diets

This makes the guarantee of the safety and authenticity of functional foods and supplements directed at this group a priority since T2DM has such a high incidence. While safety deals with ridding food products of contamination or hazardous additives, authenticity is about demonstrating the food's origin, constituents, and nutritional prerogatives. Given that food has a therapeutic outcome on glycemic control and metabolic constancy, these factors are acute in the dietary management of T2DM. The strength of consumer trust depends more on food authenticity. According to research, people with T2DM are more vulnerable to being affected by EDCs that work as mimics of the hormones and upset the balance of metabolic control (Velmurugan et al. 2017). For instance, food products contaminated with heavy metals or certain insecticides like organochlorine worsen insulin resistance and glucose intolerance (Tyagi et al. 2021; Nguyen 2023).

Food safety is equally significant because people with T2DM are more prone to inflammation and oxidative stress (Chelliah et al. 2022). Intake of contaminated food will enhance these processes and worsen side effects such as nephropathy or neuropathy (He et al. 2024). Moreover, vulnerable communities are more susceptible to alterations in exposure to harmful pollutants, which enhances the rate of diabetes (Ruiz et al. 2018).

The interdisciplinary area of foodomics connects food science to applied omics technologies as a marvelous transformer, and, specifically, results are offered by applied approaches like DNA barcoding, proteomics, and metabolomics. Such results tell us about food composition, recognize contaminants, and ensure that nutritional promises are fulfilled (Su et al. 2024; Galimberti et al. 2019). These resources are predominantly relevant to diabetes‐explicit products that must comply with strict regulations to be compliant and, expectantly, safe from adverse health issues. According to Galimberti et al. (2019), DNA barcoding is a successful method for the authentication of the botanical constituents of functional foods and dietary supplements applied in diabetes management. With such an approach, active ingredients—namely, polyphenols or bioactive peptides necessary for glycemic control (Chhikara et al. 2021)—are ensured to be present in adequate quantities.

Additionally, metabolomic profiling can identify pollutants that could exacerbate metabolic diseases by revealing biomarkers of food quality (Sébédio 2017). There has been concern that some of these environmental contaminants, including phthalates, bisphenol A (BPA), and persistent organic pollutants, exist in food chains and can potentially induce diabetes (Petrakis et al. 2017). EDCs have been implicated in T2DM etiology through interference with insulin signaling pathways and mitochondrial function (Neel and Sargis 2011). These pollutants can be identified in food compounds, and their effects on metabolic well‐being can be contained using multi‐omics approaches, as with He et al. (2024) and Zhang et al. (2024).

### 9.3 Regulation and Consumer Awareness

Despite advancements in foodomics, standards and criteria for labeling and calibration of functional foods and supplements to manage diabetes are not yet flawless (Dagar et al. 2023). Harsher laws need to be implemented so that they are suitably labeled and tested to safeguard customers (Abdel‐Tawab 2018). Customers also need to be aware of the danger of adulterated or spurious products so that they can make informed nutritional decisions (Villamiel and Méndez‐Albiñana 2022).

Employing foodomics to improve food security and authenticity methods will be an appropriate method of enhancing the quality of foods made for diabetic patients. Such methods uphold the overall objective of reducing the health hazards caused by diabetes by eliminating environmental weaknesses and proper labeling. Scientific examination and regulatory enhancements are always required to ensure the quality of functional foods in controlling T2DM.

## 10 Current Limitations in Foodomics for Diabetes Research

Standardization of methods and handling large, complex data sets are two key barriers to the application of foodomics in diabetes studies. Integration of multiple omics platforms, including proteomics, metabolomics, and genomics, to obtain consistent analysis results is problematic due to heterogeneity in sample processing, collection, and analysis protocols (Su et al. 2024; Sébédio 2017). As there is not sufficient consistency, it is difficult to contrast outcomes between studies, and outcomes are not readily replicable, lowering the practicality and reliability of foodomics (Galimberti et al. 2019). The greatest hindrance is obtaining a mechanistic understanding of diabetes dietary therapies. While multi‐omics tools have discovered promising diabetes biomarkers, there is not yet sufficient meaningful dietary advice informed by research evidence. For example, the precise mechanisms through which some bioactive compounds can influence inflammation, insulin sensitivity, and glucose metabolism remain unknown (Petrakis et al. 2017; Chhikara et al. 2021).

Dietary interference is stronger or weaker depending on specific interfaces between environmental pollutants, food components, and metabolic well‐being; hence, further research into such interactions is warranted (Velmurugan et al. 2017; He et al. 2024). Despite existing challenges, foodomics is poised to have a significant role in driving precision medicine and individualized dietary advice in the management of T2DM. With the support of multi‐omics technology, medication can be more precisely attuned to the patient's personalized genetic, metabolic, and microbiome profiles (Su et al. 2024; Galimberti et al. 2019). For the best regulator of blood glucose levels and plummeting complications, metabolomic valuation can help identify who will derive supreme benefit from an exact functional diet or supplement (Sébédio 2017; Abdel‐Tawab 2018).

Separately from T2DM, food‐based genomics for therapeutic determinations treats metabolic syndrome, obesity, and other related disorders. A new investigation has exposed that food and dietary quality play a significant role in the deterrence and management of such circumstances. This means that foodomics can assist in determining bioactive composites and metabolic paths that are responsible for improved health (Nguyen 2023; Petrakis et al. 2017). Lastly, interventions in mitigation may be followed with the assistance of foodomics coupled with environmental data, which also helps further portray the influences of pollutants on metabolic health (Ruiz et al. 2018; Tyagi et al. 2021). Regardless of ongoing molecular standards trials, foodomics is revolutionizing dietary and anticipation approaches to combat T2DM and connected metabolic disorders. To understand foodomics' full prospective as a forum for global health development discourse, additional multidisciplinary associations, data convergence, and technological progressions are obligatory.

## 11 Conclusion and Future Perspectives

To improve the management of T2DM, the current study demonstrates how foodomics can develop our understanding of the disease and offer more precise and effective dietary treatments. Foodomics is a field that unites genomes, proteomics, and metabolomics to determine biomarkers and metabolic pathways that are essential in diabetes. Enhanced glycemic control based on a person's metabolic profile and abridged risk of long‐term side effects has been achieved as a consequence of the rationalization of nutrition‐based treatment development allowed by this systems‐level strategy. The therapeutic potential of bioactive food constituents like polyphenols and dietary fiber in refining insulin sensitivity and modulating blood glucose levels has also been explored in the current investigation. These outcomes open novel opportunities for the formation of meals with functional properties that could aid in the prevention and treatment of T2DM.

New dietary methods to reduce the influence of T2DM by using microbiome‐targeting therapies have been planned by foodomics, which assists us in knowing more about the role of gut microbiota in metabolic control. Before foodomics is applied fully in the clinical management of diabetes, there are certain issues that need to be resolved, despite their auspicious applications. Among them, we need to collect huge, diverse datasets, normalize foodomics methods, and perform more in‐depth studies to reveal the molecular mechanisms underlying food–metabolic disease associations. In addition, functional foods identified by foodomics need further testing to validate their efficacy and safety before they can be used in evidence‐based therapeutic strategies for diabetes. Foodomics has vast potential beyond T2DM and could change the way a variety of metabolic diseases are treated. Precision medicine, individualized diet, and the continued evolution of foodomics technology could someday result in improved, more efficient, and preventive strategies for controlling chronic diseases if delivered within clinical contexts. Designer dietary treatments that take into consideration an individual's own genetic, metabolic, and microbiome fingerprint contain personalized nutrition, an exciting new area in foodomics.

Foodomics will develop a mainstay of personalized diabetes care as precision medicine progresses further, permitting doctors to prescribe dietary recommendations tailored to each patient's exclusive molecular signature in anticipation of being better able to accomplish blood sugar control and avoid complications. Furthermore, foodomics may lead to a cohort of microbiome‐based dietary interferences, following on from the cumulative body of work investigating the association between metabolic illness and gut bacteria. As it reduces inflammation, these treatments aim to improve insulin sensitivity to endorse a greater overall method toward diabetes management. Patients with T2DM depend on food quality and safety, two aspects in which foodomics has wider implications. In addition to staying away from risks within foodstuffs, foodomics can perceive outside risks to patients' diets in foodstuffs, such as pesticides, heavy metals, or endocrine disruptors, which would worsen the disease. Technology may assist diabetics in the future as well by ensuring that functional foods and nutritional supplements are safe and authentic. Overcoming current challenges, including the integration of multi‐omics data, standardization of analytical methods, and translating findings into therapeutic applications, will probably be the focus of future foodomics research. Foodomics can be a game changer in precision nutrition as the science continues to evolve, improving public health globally for a range of chronic diseases, including T2DM.

## Author Contributions

**Sammra Maqsood:** methodology (equal), writing – original draft (equal). **Muhammad Tayyab Arshad:** data curation (equal), writing – review and editing (equal). **Ali Ikram:** supervision (equal), validation (equal). **Hatem A. Al‐Aoh:** data curation (equal). **Kodjo Théodore Gnedeka:** project administration (equal), resources (equal).

## Disclosure

The authors have nothing to report.

## Ethics Statement

The authors have nothing to report.

## Consent

The authors have nothing to report.

## Conflicts of Interest

The authors declare no conflicts of interest.

## Data Availability

The data supporting this study's findings are available from the corresponding author upon reasonable request.

## References

[fsn371021-bib-0001] Abdel‐Tawab, M. 2018. “Do We Need Plant Food Supplements? A Critical Examination of Quality, Safety, Efficacy, and Necessity for a New Regulatory Framework.” Planta Medica 84, no. 6/7: 372–393. 29220861 10.1055/s-0043-123764

[fsn371021-bib-0002] Abdurahman, A. A. , E. E. Chaka , S. Nedjat , A. R. Dorosty , and R. Majdzadeh . 2019. “The Association of Household Food Insecurity With the Risk of Type 2 Diabetes Mellitus in Adults: A Systematic Review and Meta‐Analysis.” European Journal of Nutrition 58: 1341–1350. 29721679 10.1007/s00394-018-1705-2

[fsn371021-bib-0003] Agrawal, G. K. , A. M. Timperio , L. Zolla , V. Bansal , R. Shukla , and R. Rakwal . 2013. “Biomarker Discovery and Applications for Foods and Beverages: Proteomics to Nanoproteomics.” Journal of Proteomics 93: 74–92. 23619387 10.1016/j.jprot.2013.04.014

[fsn371021-bib-0004] Alaofè, H. , M. Mahdavimanshadi , C. Mizéhoun‐Adissoda , et al. 2024. “Precision Nutrition for Type 2 Diabetes in Benin: Leveraging Linear Goal Programming to Optimize Diets With Emphasis on Adequacy, Affordability, Accessibility, and Culture.” Frontiers in Nutrition 11: 1400594. 39176027 10.3389/fnut.2024.1400594 PMC11338894

[fsn371021-bib-0005] Alba, C. M.‐A. , M. Daya , and C. Franck . 2019. “Tart Cherries and Health: Current Knowledge and Need for a Better Understanding of the Fate of Phytochemicals in the Human Gastrointestinal Tract.” Critical Reviews in Food Science and Nutrition 59, no. 4: 626–638. 28956621 10.1080/10408398.2017.1384918

[fsn371021-bib-0006] Albu, J. B. , L. K. Heilbronn , D. E. Kelley , et al. 2010. “Metabolic Changes Following a 1‐Year Diet and Exercise Intervention in Patients With Type 2 Diabetes.” Diabetes 59, no. 3: 627–633. 20028945 10.2337/db09-1239 PMC2828653

[fsn371021-bib-0007] Andjelković, U. , and D. Josić . 2018. “Mass Spectrometry Based Proteomics as Foodomics Tool in Research and Assurance of Food Quality and Safety.” Trends in Food Science & Technology 77: 100–119.

[fsn371021-bib-0008] Andjelković, U. , M. Šrajer Gajdošik , D. Gašo‐Sokač , T. Martinović , and D. Josić . 2017. “Foodomics and Food Safety: Where We Are.” Food Technology and Biotechnology 55, no. 3: 290–307. 29089845 10.17113/ftb.55.03.17.5044 PMC5654429

[fsn371021-bib-0009] Antwi, J. 2023. “Precision Nutrition to Improve Risk Factors of Obesity and Type 2 Diabetes.” Current Nutrition Reports 12, no. 4: 679–694. 37610590 10.1007/s13668-023-00491-y PMC10766837

[fsn371021-bib-0010] Anwardeen, N. R. , K. Naja , and M. A. Elrayess . 2024. “Advancements in Precision Medicine: Multi‐Omics Approach for Tailored Metformin Treatment in Type 2 Diabetes.” Frontiers in Pharmacology 15: 1506767. 39669200 10.3389/fphar.2024.1506767 PMC11634602

[fsn371021-bib-0011] Armenteros, J. J. A. , C. Brorsson , C. H. Johansen , et al. 2024. “Multi‐Omics Analysis Reveals Drivers of Loss of β‐Cell Function After Newly Diagnosed Autoimmune Type 1 Diabetes: An INNODIA Multicenter Study.” Diabetes/Metabolism Research and Reviews 40, no. 5: e3833. 38961656 10.1002/dmrr.3833

[fsn371021-bib-0194] Arshad, M. T. , M. K. M. Ali , S. Maqsood , et al. 2025. “Dietary Phytochemicals in Cardiovascular Disease Prevention and Management: A Comprehensive Review.” Food Science & Nutrition 13, no. 9: e70872. 40909258 10.1002/fsn3.70872 PMC12406175

[fsn371021-bib-0013] Azam, M. , M. Saeed , and M. H. Ahmad . 2019. “Nuclear Magnetic Resonance Spectroscopy: A Useful Analytical Tool to Determine Different Parameters in Food Applications.” In Advances in Noninvasive Food Analysis, 123–152. CRC Press.

[fsn371021-bib-0014] Backman, M. , F. Flenkenthaler , A. Blutke , et al. 2019. “Multi‐Omics Insights Into Functional Alterations of the Liver in Insulin‐Deficient Diabetes Mellitus.” Molecular Metabolism 26: 30–44. 31221621 10.1016/j.molmet.2019.05.011 PMC6667734

[fsn371021-bib-0015] Balkir, P. , K. Kemahlioglu , and U. Yucel . 2021. “Foodomics: A New Approach in Food Quality and Safety.” Trends in Food Science & Technology 108: 49–57.

[fsn371021-bib-0016] Barnett, M. , W. Young , J. Cooney , and N. Roy . 2015. “Metabolomics and Proteomics, and What to Do With All These ‘Omes’: Insights From Nutrigenomic Investigations in New Zealand.” Lifestyle Genomics 7, no. 4–6: 274–282. 10.1159/000381349 25997469

[fsn371021-bib-0017] Ben‐Yacov, O. , and M. Rein . 2022. “Precision Nutrition for Type 2 Diabetes.” In Precision Medicine in Diabetes: A Multidisciplinary Approach to an Emerging Paradigm, 233–249. Springer International Publishing.

[fsn371021-bib-0018] Bordoni, A. , and F. Capozzi . 2014. “Foodomics for Healthy Nutrition.” Current Opinion in Clinical Nutrition & Metabolic Care 17, no. 5: 418–424. 25010544 10.1097/MCO.0000000000000089

[fsn371021-bib-0019] Borgundvaag, E. , J. Mak , and C. K. Kramer . 2021. “Metabolic Impact of Intermittent Fasting in Patients With Type 2 Diabetes Mellitus: A Systematic Review and Meta‐Analysis of Interventional Studies.” Journal of Clinical Endocrinology & Metabolism 106, no. 3: 902–911. 33319233 10.1210/clinem/dgaa926

[fsn371021-bib-0020] Braconi, D. , G. Bernardini , L. Millucci , and A. Santucci . 2018. “Foodomics for Human Health: Current Status and Perspectives.” Expert Review of Proteomics 15, no. 2: 153–164. 29271263 10.1080/14789450.2018.1421072

[fsn371021-bib-0022] Brennan, C. S. , and L. J. Cleary . 2005. “The Potential Use of Cereal (1→ 3, 1 → 4)‐β‐D‐Glucans as Functional Food Ingredients.” Journal of Cereal Science 42, no. 1: 1–13.

[fsn371021-bib-0023] Cajka, T. 2024. “Liquid Chromatography–Mass Spectrometry‐Based Metabolomics Approaches for Foodomics Research.” Current Opinion in Food Science 58: 101201.

[fsn371021-bib-0024] Canela, N. , M. Á. Rodríguez , I. Baiges , P. Nadal , and L. Arola . 2016. “Foodomics Imaging by Mass Spectrometry and Magnetic Resonance.” Electrophoresis 37, no. 13: 1748–1767. 26799681 10.1002/elps.201500494

[fsn371021-bib-0025] Capozzi, F. , and A. Bordoni . 2013. “Foodomics: A New Comprehensive Approach to Food and Nutrition.” Genes & Nutrition 8: 1–4. 22933238 10.1007/s12263-012-0310-x PMC3535000

[fsn371021-bib-0026] Caratti, A. , S. Squara , C. Bicchi , et al. 2024. “Boosting Comprehensive Two‐Dimensional Chromatography With Artificial Intelligence: Application to Food‐Omics.” TrAC Trends in Analytical Chemistry 174: 117669.

[fsn371021-bib-0027] Carter, C. J. , and R. A. Blizard . 2016. “Autism Genes Are Selectively Targeted by Environmental Pollutants Including Pesticides, Heavy Metals, Bisphenol A, Phthalates and Many Others in Food, Cosmetics or Household Products.” Neurochemistry International 101: 83–109. 10.1016/j.neuint.2016.10.011 27984170

[fsn371021-bib-0028] Castro‐Muñoz, R. , M. Correa‐Delgado , R. Córdova‐Almeida , et al. 2022. “Natural Sweeteners: Sources, Extraction and Current Uses in Foods and Food Industries.” Food Chemistry 370: 130991. 34509947 10.1016/j.foodchem.2021.130991

[fsn371021-bib-0029] Cavalli, M. , K. Diamanti , Y. Dang , et al. 2021. “The Thioesterase ACOT1 as a Regulator of Lipid Metabolism in Type 2 Diabetes Detected in a Multi‐Omics Study of Human Liver.” Omics: A Journal of Integrative Biology 25, no. 10: 652–659. 34520261 10.1089/omi.2021.0093 PMC8812507

[fsn371021-bib-0030] Chatterjee, S. , K. Khunti , and M. J. Davies . 2017. “Type 2 Diabetes.” Lancet 389, no. 10085: 2239–2251. 28190580 10.1016/S0140-6736(17)30058-2

[fsn371021-bib-0032] Chelliah, R. , E. Banan‐MwineDaliri , I. Khan , et al. 2022. “A Review on the Application of Bioinformatics Tools in Food Microbiome Studies.” Briefings in Bioinformatics 23, no. 2: bbac007. 35189636 10.1093/bib/bbac007

[fsn371021-bib-0033] Chen, D. , X. Zhao , Z. Sui , et al. 2020. “A Multi‐Omics Investigation of the Molecular Characteristics and Classification of Six Metabolic Syndrome Relevant Diseases.” Theranostics 10, no. 5: 2029–2046. 32089734 10.7150/thno.41106 PMC7019171

[fsn371021-bib-0186] Cheng, X. , J. Huang , H. Li , et al. 2024. “Quercetin: A Promising Therapy for Diabetic Encephalopathy Through Inhibition of Hippocampal Ferroptosis.” Phytomedicine 126: 154887. 10.1016/j.phymed.2023.154887. 38377720

[fsn371021-bib-0036] Chevalier, N. , and P. Fénichel . 2015. “Endocrine Disruptors: New Players in the Pathophysiology of Type 2 Diabetes?” Diabetes & Metabolism 41, no. 2: 107–115. 25454091 10.1016/j.diabet.2014.09.005

[fsn371021-bib-0037] Chhikara, N. , A. Kaur , S. Mann , M. K. Garg , S. A. Sofi , and A. Panghal . 2021. “Bioactive Compounds, Associated Health Benefits and Safety Considerations of *Moringa oleifera* L.: An Updated Review.” Nutrition & Food Science 51, no. 2: 255–277.

[fsn371021-bib-0038] Chung, H. J. , J. H. Sim , T. S. Min , and H. K. Choi . 2018. “Metabolomics and Lipidomics Approaches in the Science of Probiotics: A Review.” Journal of Medicinal Food 21, no. 11: 1086–1095. 30004273 10.1089/jmf.2017.4175

[fsn371021-bib-0039] Corsaro, C. , N. Cicero , D. Mallamace , et al. 2016. “HR‐MAS and NMR Towards Foodomics.” Food Research International 89: 1085–1094.

[fsn371021-bib-0040] Dagar, M. , P. Kumari , A. M. W. Mirza , et al. 2023. “The Hidden Threat: Endocrine Disruptors and Their Impact on Insulin Resistance.” Cureus 15, no. 10: e47282. 38021644 10.7759/cureus.47282 PMC10656111

[fsn371021-bib-0041] Damarov, I. S. , E. E. Korbolina , E. Y. Rykova , and T. I. Merkulova . 2024. “Multi‐Omics Analysis Revealed the rSNPs Potentially Involved in T2D Pathogenic Mechanism and Metformin Response.” International Journal of Molecular Sciences 25, no. 17: 9297. 39273245 10.3390/ijms25179297 PMC11394919

[fsn371021-bib-0042] Ďásková, N. , I. Modos , M. Krbcová , et al. 2023. “Multi‐Omics Signatures in New‐Onset Diabetes Predict Metabolic Response to Dietary Inulin: Findings From an Observational Study Followed by an Interventional Trial.” Nutrition & Diabetes 13, no. 1: 7. 37085526 10.1038/s41387-023-00235-5 PMC10121613

[fsn371021-bib-0043] de Toro‐Martín, J. , B. J. Arsenault , J. P. Després , and M. C. Vohl . 2017. “Precision Nutrition: A Review of Personalized Nutritional Approaches for the Prevention and Management of Metabolic Syndrome.” Nutrients 9, no. 8: 913. 28829397 10.3390/nu9080913 PMC5579706

[fsn371021-bib-0044] Drincic, A. T. , J. T. Knezevich , and P. Akkireddy . 2017. “Nutrition and Hyperglycemia Management in the Inpatient Setting (Meals on Demand, Parenteral, or Enteral Nutrition).” Current Diabetes Reports 17: 1–12. 28664252 10.1007/s11892-017-0882-3

[fsn371021-bib-0045] Eddy, S. , L. H. Mariani , and M. Kretzler . 2020. “Integrated Multi‐Omics Approaches to Improve Classification of Chronic Kidney Disease.” Nature Reviews Nephrology 16, no. 11: 657–668. 32424281 10.1038/s41581-020-0286-5

[fsn371021-bib-0046] Ellett, S. , I. R. Ferguson , S. Zhu , et al. 2013. “13 Foodomics to Study Efficacy of Human Dietary Interventions.” In Nutrigenomics and Nutrigenetics in Functional Foods and Personalized Nutrition, 269. CRC Press.

[fsn371021-bib-0047] Fan, K. , and M. Zhang . 2019. “Recent Developments in the Food Quality Detected by Non‐Invasive Nuclear Magnetic Resonance Technology.” Critical Reviews in Food Science and Nutrition 59, no. 14: 2202–2213. 29451810 10.1080/10408398.2018.1441124

[fsn371021-bib-0048] Faulkner, A. , Z. Dang , E. Avolio , et al. 2020. “Multi‐Omics Analysis of Diabetic Heart Disease in the Db/Db Model Reveals Potential Targets for Treatment by a Longevity‐Associated Gene.” Cells 9, no. 5: 1283. 32455800 10.3390/cells9051283 PMC7290798

[fsn371021-bib-0049] Fénichel, P. , and N. Chevalier . 2017. “Environmental Endocrine Disruptors: New Diabetogens?” Comptes Rendus Biologies 340, no. 9–10: 446–452. 28826789 10.1016/j.crvi.2017.07.003

[fsn371021-bib-0050] Fernández‐Ochoa, Á. , F. J. Leyva‐Jiménez , M. De la Luz Cádiz‐Gurrea , S. Pimentel‐Moral , and A. Segura‐Carretero . 2021. “The Role of High‐Resolution Analytical Techniques in the Development of Functional Foods.” International Journal of Molecular Sciences 22, no. 6: 3220. 33809986 10.3390/ijms22063220 PMC8004826

[fsn371021-bib-0052] Fitipaldi, H. , M. I. McCarthy , J. C. Florez , and P. W. Franks . 2018. “A Global Overview of Precision Medicine in Type 2 Diabetes.” Diabetes 67, no. 10: 1911–1922. 30237159 10.2337/dbi17-0045 PMC6152339

[fsn371021-bib-0192] Fu, B. , W. Liu , Y. Wang , et al. 2024. “Design and Synthesis of Thiourea‐Conjugating Organic Arsenic D‐Glucose with Anticancer Activities.” Molecules 29, no. 12: 2850. 10.3390/molecules29122850. 38930915 PMC11206549

[fsn371021-bib-0053] Fung, F. , H. S. Wang , and S. Menon . 2018. “Food Safety in the 21st Century.” Biomedical Journal 41, no. 2: 88–95. 29866604 10.1016/j.bj.2018.03.003 PMC6138766

[fsn371021-bib-0054] Galimberti, A. , M. Casiraghi , I. Bruni , et al. 2019. “From DNA Barcoding to Personalized Nutrition: The Evolution of Food Traceability.” Current Opinion in Food Science 28: 41–48.

[fsn371021-bib-0055] Gallo, M. , and P. Ferranti . 2016. “The Evolution of Analytical Chemistry Methods in Foodomics.” Journal of Chromatography A 1428: 3–15. 26363946 10.1016/j.chroma.2015.09.007

[fsn371021-bib-0056] Garg, R. , and D. Heber . 2024. “Precision Nutrition in Diabetes.” In Precision Nutrition, 201–214. Academic Press.

[fsn371021-bib-0057] Geng, L. , M. Li , G. Zhang , and L. Ye . 2022. “Barley: A Potential Cereal for Producing Healthy and Functional Foods.” Food Quality and Safety 6: fyac012.

[fsn371021-bib-0058] Ghallab, D. S. , D. A. Ghareeb , and D. A. Goda . 2024. “Integrative Metabolomics and Chemometrics Depict the Metabolic Alterations of Differently Processed Red Kidney Beans ( *Phaseolus vulgaris* L.) and in Relation to In‐Vitro Anti‐Diabetic Efficacy.” Food Research International 192: 114786. 39147477 10.1016/j.foodres.2024.114786

[fsn371021-bib-0059] Giacco, R. , B. De Giulio , M. Vitale , and R. Cozzolino . 2013. “Functional Foods: Can Food Technology Help in the Prevention and Treatment of Diabetes?” Food and Nutrition Sciences 04: 827–837.

[fsn371021-bib-0060] Gilbert‐López, B. , A. Valdés , T. Acunha , V. García‐Cañas , C. Simó , and A. Cifuentes . 2017. “Foodomics: LC and LC‐MS‐Based Omics Strategies in Food Science and Nutrition.” In Liquid Chromatography, 267–299. Elsevier.

[fsn371021-bib-0061] Gloyn, A. L. , and D. J. Drucker . 2018. “Precision Medicine in the Management of Type 2 Diabetes.” Lancet Diabetes & Endocrinology 6, no. 11: 891–900. 29699867 10.1016/S2213-8587(18)30052-4

[fsn371021-bib-0062] Godman, B. , D. Basu , Y. Pillay , et al. 2020. “Review of Ongoing Activities and Challenges to Improve the Care of Patients With Type 2 Diabetes Across Africa and the Implications for the Future.” Frontiers in Pharmacology 11: 108. 32265688 10.3389/fphar.2020.00108 PMC7098994

[fsn371021-bib-0063] González‐Sálamo, J. , D. A. Varela‐Martínez , M. Á. González‐Curbelo , and J. Hernández‐Borges . 2021. “The Role of Chromatographic and Electromigration Techniques in Foodomics.” In Separation Techniques Applied to Omics Sciences: From Principles to Relevant Applications, 31–49. Springer Nature. 10.1007/978-3-030-77252-9_3 34628626

[fsn371021-bib-0064] Guo, H. , H. Wu , A. Sajid , and Z. Li . 2022. “Whole Grain Cereals: The Potential Roles of Functional Components in Human Health.” Critical Reviews in Food Science and Nutrition 62, no. 30: 8388–8402. 34014123 10.1080/10408398.2021.1928596

[fsn371021-bib-0065] Gupta, R. , P. Kumar , N. Fahmi , et al. 2020. “Endocrine Disruption and Obesity: A Current Review on Environmental Obesogens.” Current Research in Green and Sustainable Chemistry 3: 100009.

[fsn371021-bib-0068] Hasin, Y. , M. Seldin , and A. Lusis . 2017. “Multi‐Omics Approaches to Disease.” Genome Biology 18: 1–15. 28476144 10.1186/s13059-017-1215-1 PMC5418815

[fsn371021-bib-0069] Hatzakis, E. 2019. “Nuclear Magnetic Resonance (NMR) Spectroscopy in Food Science: A Comprehensive Review.” Comprehensive Reviews in Food Science and Food Safety 18, no. 1: 189–220. 33337022 10.1111/1541-4337.12408

[fsn371021-bib-0070] Haverinen, E. , M. F. Fernandez , V. Mustieles , and H. Tolonen . 2021. “Metabolic Syndrome and Endocrine Disrupting Chemicals: An Overview of Exposure and Health Effects.” International Journal of Environmental Research and Public Health 18, no. 24: 13047. 34948652 10.3390/ijerph182413047 PMC8701112

[fsn371021-bib-0071] He, K. , R. Chen , S. Xu , et al. 2024. “Environmental Endocrine Disruptor‐Induced Mitochondrial Dysfunction: A Potential Mechanism Underlying Diabetes and Its Complications.” Frontiers in Endocrinology 15: 1422752. 39211449 10.3389/fendo.2024.1422752 PMC11357934

[fsn371021-bib-0072] He, M. , C. P. Tan , Y. Liu , and Y. J. Xu . 2021. “Foodomics: A New Perspective on Gut Probiotics Nutrition and Health Research.” Current Opinion in Food Science 41: 146–151.

[fsn371021-bib-0073] Hectors, T. L. M. , C. Vanparys , K. Van Der Ven , et al. 2011. “Environmental Pollutants and Type 2 Diabetes: A Review of Mechanisms That Can Disrupt Beta Cell Function.” Diabetologia 54: 1273–1290. 21442161 10.1007/s00125-011-2109-5

[fsn371021-bib-0074] Henneke, L. , K. Schlicht , N. A. Andreani , et al. 2022. “A Dietary Carbohydrate–Gut Parasutterella–Human Fatty Acid Biosynthesis Metabolic Axis in Obesity and Type 2 Diabetes.” Gut Microbes 14, no. 1: 2057778. 35435797 10.1080/19490976.2022.2057778 PMC9037427

[fsn371021-bib-0075] Herrero, M. , C. Simó , V. García‐Cañas , E. Ibáñez , and A. Cifuentes . 2012. “Foodomics: MS‐Based Strategies in Modern Food Science and Nutrition.” Mass Spectrometry Reviews 31, no. 1: 49–69. 21374694 10.1002/mas.20335

[fsn371021-bib-0076] Hosomi, K. , M. Saito , J. Park , et al. 2022. “Oral Administration of *Blautia wexlerae* Ameliorates Obesity and Type 2 Diabetes via Metabolic Remodeling of the Gut Microbiota.” Nature Communications 13, no. 1: 4477. 10.1038/s41467-022-32015-7 PMC9388534 35982037

[fsn371021-bib-0078] Ibáñez, C. , C. Simó , V. García‐Cañas , A. Cifuentes , and M. Castro‐Puyana . 2013. “Metabolomics, Peptidomics and Proteomics Applications of Capillary Electrophoresis‐Mass Spectrometry in Foodomics: A Review.” Analytica Chimica Acta 802: 1–13. 24176500 10.1016/j.aca.2013.07.042

[fsn371021-bib-0079] Ibáñez, E. , and A. Cifuentes . 2014. “Foodomics: Food Science and Nutrition in the Postgenomic Era.” In Comprehensive Analytical Chemistry, vol. 64, 395–440. Elsevier.

[fsn371021-bib-0080] Idehen, E. , W. Wang , and S. Sang . 2020. “Health Benefits of Barley for Diabetes.” Journal of Food Bioactives 12: 76–86.

[fsn371021-bib-0081] Jaacks, L. M. , K. R. Siegel , U. P. Gujral , and K. V. Narayan . 2016. “Type 2 Diabetes: A 21st Century Epidemic.” Best Practice & Research Clinical Endocrinology & Metabolism 30, no. 3: 331–343. 27432069 10.1016/j.beem.2016.05.003

[fsn371021-bib-0082] Janda, K. , A. Orłowska , K. Watychowicz , and K. Jakubczyk . 2019. “The Role of Oat Products in the Prevention and Therapy of Type 2 Diabetes, Hypercholesterolemia and Obesity.” Pomeranian Journal of Life Sciences 65, no. 4: 30–36.

[fsn371021-bib-0083] Jiang, F. , L. Yuan , N. Shu , W. Wang , Y. Liu , and Y. J. Xu . 2020. “Foodomics Revealed the Effects of Extract Methods on the Composition and Nutrition of Peanut Oil.” Journal of Agricultural and Food Chemistry 68, no. 4: 1147–1156. 31917573 10.1021/acs.jafc.9b06819

[fsn371021-bib-0084] Kautzky‐Willer, A. , M. Leutner , and J. Harreiter . 2023. “Sex Differences in Type 2 Diabetes.” Diabetologia 66, no. 6: 986–1002. 36897358 10.1007/s00125-023-05891-x PMC10163139

[fsn371021-bib-0085] Keijer, J. , X. Escoté , S. Galmés , et al. 2024. “Omics Biomarkers and an Approach for Their Practical Implementation to Delineate Health Status for Personalized Nutrition Strategies.” Critical Reviews in Food Science and Nutrition 64, no. 23: 8279–8307. 37077157 10.1080/10408398.2023.2198605

[fsn371021-bib-0086] Khalil, W. J. , M. Akeblersane , A. S. Khan , A. S. M. Moin , and A. E. Butler . 2023. “Environmental Pollution and the Risk of Developing Metabolic Disorders: Obesity and Diabetes.” International Journal of Molecular Sciences 24, no. 10: 8870. 37240215 10.3390/ijms24108870 PMC10219141

[fsn371021-bib-0088] Kuo, C. C. , K. Moon , K. A. Thayer , and A. Navas‐Acien . 2013. “Environmental Chemicals and Type 2 Diabetes: An Updated Systematic Review of the Epidemiologic Evidence.” Current Diabetes Reports 13: 831–849. 24114039 10.1007/s11892-013-0432-6 PMC4327889

[fsn371021-bib-0089] Kupai, K. , T. Várkonyi , S. Török , et al. 2022. “Recent Progress in the Diagnosis and Management of Type 2 Diabetes Mellitus in the Era of COVID‐19 and Single Cell Multi‐Omics Technologies.” Life 12, no. 8: 1205. 36013384 10.3390/life12081205 PMC9409806

[fsn371021-bib-0090] Laghi, L. , G. Picone , and F. Capozzi . 2014. “Nuclear Magnetic Resonance for Foodomics Beyond Food Analysis.” TrAC Trends in Analytical Chemistry 59: 93–102.

[fsn371021-bib-0091] Lee, D. H. , M. Porta , D. R. Jacobs Jr. , and L. N. Vandenberg . 2014. “Chlorinated Persistent Organic Pollutants, Obesity, and Type 2 Diabetes.” Endocrine Reviews 35, no. 4: 557–601. 24483949 10.1210/er.2013-1084 PMC5393257

[fsn371021-bib-0092] LeVatte, M. , A. H. Keshteli , P. Zarei , and D. S. Wishart . 2022. “Applications of Metabolomics to Precision Nutrition.” Lifestyle Genomics 15, no. 1: 1–9. 34518463 10.1159/000518489

[fsn371021-bib-0191] Li, A. N. , J. J. Chen , Q. Q. Li , et al. 2019. “Alpha‐glucosidase inhibitor 1‐Deoxynojirimycin promotes beige remodeling of 3T3‐L1 preadipocytes via activating AMPK.” Biochemical and Biophysical Research Communications 509, no. 4: 1001–1007. 10.1016/j.bbrc.2019.01.023. 30654939

[fsn371021-bib-0187] Li, W. , X. Liu , Z. Liu , et al. 2024. “The Signaling Pathways of Selected Traditional Chinese Medicine Prescriptions and Their Metabolites in the Treatment of Diabetic Cardiomyopathy: A Review.” Frontiers in Pharmacology 15: 1416403. 10.3389/fphar.2024.1416403. 39021834 PMC11251973

[fsn371021-bib-0095] Li, W. , Z. Wu , Y. Xu , et al. 2023. “Emerging LC‐MS/MS‐Based Molecular Networking Strategy Facilitates Foodomics to Assess the Function, Safety, and Quality of Foods: Recent Trends and Future Perspectives.” Trends in Food Science & Technology 139: 104114.

[fsn371021-bib-0097] Lima, J. E. , N. C. Moreira , and E. T. Sakamoto‐Hojo . 2022. “Mechanisms Underlying the Pathophysiology of Type 2 Diabetes: From Risk Factors to Oxidative Stress, Metabolic Dysfunction, and Hyperglycemia.” Mutation Research‐Genetic Toxicology and Environmental Mutagenesis 874: 503437. 35151421 10.1016/j.mrgentox.2021.503437

[fsn371021-bib-0098] Liu, D. , Q. Shi , C. Liu , Q. Sun , and X. Zeng . 2023. “Effects of Endocrine‐Disrupting Heavy Metals on Human Health.” Toxics 11, no. 4: 322. 37112549 10.3390/toxics11040322 PMC10147072

[fsn371021-bib-0099] Liu, J. , S. Liu , Z. Yu , X. Qiu , R. Jiang , and W. Li . 2022. “Uncovering the Gene Regulatory Network of Type 2 Diabetes Through Multi‐Omic Data Integration.” Journal of Translational Medicine 20, no. 1: 604. 36527108 10.1186/s12967-022-03826-5 PMC9756634

[fsn371021-bib-0100] López‐Moreno, A. , I. Acuña , A. Torres‐Sánchez , et al. 2021. “Next Generation Probiotics for Neutralizing Obesogenic Effects: Taxa Culturing Searching Strategies.” Nutrients 13, no. 5: 1617. 34065873 10.3390/nu13051617 PMC8151043

[fsn371021-bib-0101] Lorber, D. 2014. “Importance of Cardiovascular Disease Risk Management in Patients With Type 2 Diabetes Mellitus.” Diabetes, Metabolic Syndrome and Obesity: Targets and Therapy 7: 169–183. 24920930 10.2147/DMSO.S61438 PMC4043722

[fsn371021-bib-0193] Maqsood, S. , N. S. Basher , M. T. Arshad , et al. 2025. “Anthocyanins From Sweet Potatoes (*Ipomoea batatas*): Bioavailability, Mechanisms of Action, and Therapeutic Potential in Diabetes and Metabolic Disorders.” Food Science & Nutrition 13, no. 9: e70895. 40918166 10.1002/fsn3.70895 PMC12409302

[fsn371021-bib-0102] Meneilly, G. S. , and T. Elliott . 1999. “Metabolic Alterations in Middle‐Aged and Elderly Obese Patients With Type 2 Diabetes.” Diabetes Care 22, no. 1: 112–118. 10333911 10.2337/diacare.22.1.112

[fsn371021-bib-0103] Meng Jia, M. J. , N. B. Niu Bing , G. S. Gu ShuQing , D. X. Deng XiaoJun , and F. Z. Fang Zhen . 2019. “Application of Proteomics Technology Based on Liquid Chromatography‐Mass Spectrometry in Food Identification.” Journal of Food Safety and Quality 10, no. 4: 998–1003.

[fsn371021-bib-0104] Merino, J. 2022. “Precision Nutrition in Diabetes: When Population‐Based Dietary Advice Gets Personal.” Diabetologia 65, no. 11: 1839–1848. 35593923 10.1007/s00125-022-05721-6

[fsn371021-bib-0105] Misra, B. B. , and A. Misra . 2020. “The Chemical Exposome of Type 2 Diabetes Mellitus: Opportunities and Challenges in the Omics Era.” Diabetes & Metabolic Syndrome: Clinical Research & Reviews 14, no. 1: 23–38. 10.1016/j.dsx.2019.12.001 31838434

[fsn371021-bib-0106] Montero, L. , and M. Herrero . 2019. “Two‐Dimensional Liquid Chromatography Approaches in Foodomics–a Review.” Analytica Chimica Acta 1083: 1–18. 31493799 10.1016/j.aca.2019.07.036

[fsn371021-bib-0107] Mortazavi, B. J. , and R. Gutierrez‐Osuna . 2023. “A Review of Digital Innovations for Diet Monitoring and Precision Nutrition.” Journal of Diabetes Science and Technology 17, no. 1: 217–223. 34467803 10.1177/19322968211041356 PMC9846399

[fsn371021-bib-0109] Mudumbi, J. B. N. , S. K. O. Ntwampe , L. Mekuto , T. Matsha , and E. F. Itoba‐Tombo . 2018. “The Role of Pollutants in Type 2 Diabetes Mellitus (T2D) and Their Prospective Impact on Phytomedicinal Treatment Strategies.” Environmental Monitoring and Assessment 190: 1–23. 10.1007/s10661-018-6634-2 29610974

[fsn371021-bib-0110] Muguruma, Y. , M. Nunome , and K. Inoue . 2022. “A Review on the Foodomics Based on Liquid Chromatography Mass Spectrometry.” Chemical and Pharmaceutical Bulletin 70, no. 1: 12–18. 34980727 10.1248/cpb.c21-00765

[fsn371021-bib-0111] Mussap, M. , A. Noto , C. Piras , L. Atzori , and V. Fanos . 2021. “Slotting Metabolomics Into Routine Precision Medicine.” Expert Review of Precision Medicine and Drug Development 6, no. 3: 173–187.

[fsn371021-bib-0112] Neel, B. A. , and R. M. Sargis . 2011. “The Paradox of Progress: Environmental Disruption of Metabolism and the Diabetes Epidemic.” Diabetes 60, no. 7: 1838–1848. 21709279 10.2337/db11-0153 PMC3121438

[fsn371021-bib-0113] Nelson, K. , L. Stojanovska , T. Vasiljevic , and M. Mathai . 2013. “Germinated Grains: A Superior Whole Grain Functional Food?” Canadian Journal of Physiology and Pharmacology 91, no. 6: 429–441. 23746040 10.1139/cjpp-2012-0351

[fsn371021-bib-0114] Nguyen, H. D. 2023. “An Evaluation of the Effects of Mixed Heavy Metals on Prediabetes and Type 2 Diabetes: Epidemiological and Toxicogenomic Analysis.” Environmental Science and Pollution Research 30, no. 34: 82437–82457. 37326729 10.1007/s11356-023-28037-3

[fsn371021-bib-0115] Ortea, I. 2022. “Foodomics in Health: Advanced Techniques for Studying the Bioactive Role of Foods.” TrAC Trends in Analytical Chemistry 150: 116589.

[fsn371021-bib-0116] Papalou, O. , E. A. Kandaraki , G. Papadakis , and E. Diamanti‐Kandarakis . 2019. “Endocrine Disrupting Chemicals: An Occult Mediator of Metabolic Disease.” Frontiers in Endocrinology 10: 112. 30881345 10.3389/fendo.2019.00112 PMC6406073

[fsn371021-bib-0117] Pellegrini, P. , P. Lemasson , L. Rastrelli , and M. D'Elia . 2024. “Effectiveness of Ketogenic Therapy in Patients With Obesity and Diabetes: A Narrative Review.” Exploration of Foods and Foodomics 2, no. 4: 313–325.

[fsn371021-bib-0189] Peng, Q. , H. Zhang , and Z. Li . 2025. “KAT2A‐Mediated H3K79 Succinylation Promotes Ferroptosis in Diabetic Nephropathy by Regulating SAT2.” Life Sciences 376: 123746. 10.1016/j.lfs.2025.123746. 40409584

[fsn371021-bib-0118] Petrakis, D. , L. Vassilopoulou , C. Mamoulakis , et al. 2017. “Endocrine Disruptors Leading to Obesity and Related Diseases.” International Journal of Environmental Research and Public Health 14, no. 10: 1282. 29064461 10.3390/ijerph14101282 PMC5664782

[fsn371021-bib-0119] Picariello, G. , G. Mamone , F. Addeo , and P. Ferranti . 2012. “Novel Mass Spectrometry‐Based Applications of the ‘omic’ Sciences in Food Technology and Biotechnology.” Food Technology and Biotechnology 50, no. 3: 286.

[fsn371021-bib-0120] Pigsborg, K. , and F. Magkos . 2022. “Metabotyping for Precision Nutrition and Weight Management: Hype or Hope?” Current Nutrition Reports 11, no. 2: 117–123. 35025088 10.1007/s13668-021-00392-y

[fsn371021-bib-0123] Ramos‐Lopez, O. 2024. “Genotype‐Based Precision Nutrition Strategies for the Prediction and Clinical Management of Type 2 Diabetes Mellitus.” World Journal of Diabetes 15, no. 2: 142–153. 38464367 10.4239/wjd.v15.i2.142 PMC10921165

[fsn371021-bib-0190] Ren, W. , Y. Xia , S. Chen , et al. 2019. “Glutamine Metabolism in Macrophages: A Novel Target for Obesity/Type 2 Diabetes.” Advances in Nutrition (Bethesda, Md.) 10, no. 2: 321–330. 10.1093/advances/nmy084. 30753258 PMC6416106

[fsn371021-bib-0124] Ren, X. , and X. Li . 2019. “Advances in Research on Diabetes by Human Nutriomics.” International Journal of Molecular Sciences 20, no. 21: 5375. 31671732 10.3390/ijms20215375 PMC6861882

[fsn371021-bib-0125] Robertson, S. , E. D. Clarke , M. Gómez‐Martín , V. Cross , C. E. Collins , and J. Stanford . 2024. “Do Precision and Personalised Nutrition Interventions Improve Risk Factors in Adults With Prediabetes or Metabolic Syndrome? A Systematic Review of Randomised Controlled Trials.” Nutrients 16, no. 10: 1479. 38794717 10.3390/nu16101479 PMC11124316

[fsn371021-bib-0126] Ruiz, D. , M. Becerra , J. S. Jagai , K. Ard , and R. M. Sargis . 2018. “Disparities in Environmental Exposures to Endocrine‐Disrupting Chemicals and Diabetes Risk in Vulnerable Populations.” Diabetes Care 41, no. 1: 193–205. 29142003 10.2337/dc16-2765 PMC5741159

[fsn371021-bib-0128] Santos, M. C. B. , L. R. da Silva Lima , C. T. dos Santos D'Almeida , et al. 2022. “Foodomics in Wheat Flour Reveals Phenolic Profile of Different Genotypes and Technological Qualities.” LWT—Food Science and Technology 153: 112519.

[fsn371021-bib-0129] Sargis, R. M. , and R. A. Simmons . 2019. “Environmental Neglect: Endocrine Disruptors as Underappreciated but Potentially Modifiable Diabetes Risk Factors.” Diabetologia 62: 1811–1822. 31451869 10.1007/s00125-019-4940-z PMC7462102

[fsn371021-bib-0130] Schulz, M. C. , and R. M. Sargis . 2021. “Inappropriately Sweet: Environmental Endocrine‐Disrupting Chemicals and the Diabetes Pandemic.” Advances in Pharmacology 92: 419–456. 34452693 10.1016/bs.apha.2021.04.002 PMC8714029

[fsn371021-bib-0131] Sébédio, J. L. 2017. “Metabolomics, Nutrition, and Potential Biomarkers of Food Quality, Intake, and Health Status.” In Advances in Food and Nutrition Research, vol. 82, 83–116. Academic Press. 28427537 10.1016/bs.afnr.2017.01.001

[fsn371021-bib-0132] Seuring, T. , O. Archangelidi , and M. Suhrcke . 2015. “The Economic Costs of Type 2 Diabetes: A Global Systematic Review.” PharmacoEconomics 33: 811–831. 25787932 10.1007/s40273-015-0268-9 PMC4519633

[fsn371021-bib-0133] Sha, Q. , J. Lyu , M. Zhao , H. Li , M. Guo , and Q. Sun . 2020. “Multi‐Omics Analysis of Diabetic Nephropathy Reveals Potential New Mechanisms and Drug Targets.” Frontiers in Genetics 11: 616435. 33362869 10.3389/fgene.2020.616435 PMC7759603

[fsn371021-bib-0134] Shamanna, P. , S. Joshi , L. Shah , et al. 2021. “Type 2 Diabetes Reversal With Digital Twin Technology‐Enabled Precision Nutrition and Staging of Reversal: A Retrospective Cohort Study.” Clinical Diabetes and Endocrinology 7: 1–8. 34776010 10.1186/s40842-021-00134-7 PMC8591797

[fsn371021-bib-0135] Shamanna, P. , B. Saboo , S. Damodharan , et al. 2020. “Reducing HbA1c in Type 2 Diabetes Using Digital Twin Technology‐Enabled Precision Nutrition: A Retrospective Analysis.” Diabetes Therapy 11: 2703–2714. 32975712 10.1007/s13300-020-00931-w PMC7547935

[fsn371021-bib-0136] Shen, Z. , Z. Y. Li , M. T. Yu , K. L. Tan , and S. Chen . 2023. “Metabolic Perspective of Astrocyte Dysfunction in Alzheimer's Disease and Type 2 Diabetes Brains.” Biomedicine & Pharmacotherapy 158: 114206. 36916433 10.1016/j.biopha.2022.114206

[fsn371021-bib-0137] Shvachko, N. A. , I. G. Loskutov , T. V. Semilet , V. S. Popov , O. N. Kovaleva , and A. V. Konarev . 2021. “Bioactive Components in Oat and Barley Grain as a Promising Breeding Trend for Functional Food Production.” Molecules 26, no. 8: 2260. 33919686 10.3390/molecules26082260 PMC8069901

[fsn371021-bib-0138] Si, W. , Y. Zhang , X. Li , Y. Du , and Q. Xu . 2021. “Understanding the Functional Activity of Polyphenols Using Omics‐Based Approaches.” Nutrients 13, no. 11: 3953. 34836207 10.3390/nu13113953 PMC8625961

[fsn371021-bib-0139] Sidhu, J. S. , Y. Kabir , and F. G. Huffman . 2007. “Functional Foods From Cereal Grains.” International Journal of Food Properties 10, no. 2: 231–244.

[fsn371021-bib-0141] Sikand, G. , P. Kris‐Etherton , and N. M. Boulos . 2015. “Impact of Functional Foods on Prevention of Cardiovascular Disease and Diabetes.” Current Cardiology Reports 17: 1–16. 25899657 10.1007/s11886-015-0593-9

[fsn371021-bib-0142] Singh, N. , R. Barthwal , A. Negi , et al. 2024. “Foodomics: Futuristic Omic Strategies to Assess the Impact of Food Nutrients on Human Health and Gut Microbiome.” International Journal of Food Science & Technology 59: 4194–4212.

[fsn371021-bib-0143] Song, X. , X. Zhang , C. Ma , X. Hu , and F. Chen . 2022. “Rediscovering the Nutrition of Whole Foods: The Emerging Role of Gut Microbiota.” Current Opinion in Food Science 48: 100908.

[fsn371021-bib-0144] Srour, B. , L. K. Fezeu , E. Kesse‐Guyot , et al. 2020. “Ultraprocessed Food Consumption and Risk of Type 2 Diabetes Among Participants of the NutriNet‐Santé Prospective Cohort.” JAMA Internal Medicine 180, no. 2: 283–291. 31841598 10.1001/jamainternmed.2019.5942 PMC6990737

[fsn371021-bib-0145] Steinbrenner, H. , L. H. Duntas , and M. P. Rayman . 2022. “The Role of Selenium in Type‐2 Diabetes Mellitus and Its Metabolic Comorbidities.” Redox Biology 50: 102236. 35144052 10.1016/j.redox.2022.102236 PMC8844812

[fsn371021-bib-0148] Su, G. , C. Yu , S. Liang , W. Wang , and H. Wang . 2024. “Multi‐Omics in Food Safety and Authenticity in Terms of Food Components.” Food Chemistry 437: 137943. 37948800 10.1016/j.foodchem.2023.137943

[fsn371021-bib-0188] Su, M. , T. Tang , W. Tang , Y. Long , L. Wang , and M. Liu . 2023. “Astragalus Improves Intestinal Barrier Function and Immunity by Acting on Intestinal Microbiota to Treat T2DM: A Research Review.” Frontiers in Immunology 14: 1243834. 10.3389/fimmu.2023.1243834. 37638043 PMC10450032

[fsn371021-bib-0149] Tayanloo‐Beik, A. , P. P. Roudsari , M. Rezaei‐Tavirani , et al. 2021. “Diabetes and Heart Failure: Multi‐Omics Approaches.” Frontiers in Physiology 12: 705424. 34421642 10.3389/fphys.2021.705424 PMC8378451

[fsn371021-bib-0150] That, L. F. L. N. , B. Xu , and J. Pandohee . 2022. “Could Foodomics Hold the Key to Unlocking the Role of Prebiotics in Gut Microbiota and Immunity?” Current Opinion in Food Science 48: 100920.

[fsn371021-bib-0151] Tiwari, A. , P. Rathor , P. K. Trivedi , and R. Ch . 2023. “Multi‐Omics Reveal Interplay Between Circadian Dysfunction and Type 2 Diabetes.” Biology 12, no. 2: 301. 36829576 10.3390/biology12020301 PMC9953493

[fsn371021-bib-0152] Tuncay, C. , and M. C. Ergoren . 2020. “A Systematic Review of Precision Nutrition and Mediterranean Diet: A Personalized Nutrition Approaches for Prevention and Management of Obesity Related Disorders.” Clinical Nutrition ESPEN 38: 61–64. 32690178 10.1016/j.clnesp.2020.04.005

[fsn371021-bib-0153] Tyagi, S. , B. K. Mishra , T. Sharma , et al. 2021. “Level of Organochlorine Pesticide in Prediabetic and Newly Diagnosed Diabetes Mellitus Patients With Varying Degree of Glucose Intolerance and Insulin Resistance Among North Indian Population.” Diabetes & Metabolism Journal 45, no. 4: 558–568. 33440917 10.4093/dmj.2020.0093 PMC8369217

[fsn371021-bib-0154] Valdés, A. , G. Alvarez‐Rivera , B. Socas‐Rodríguez , M. Herrero , E. Ibanez , and A. Cifuentes . 2021. “Foodomics: Analytical Opportunities and Challenges.” Analytical Chemistry 94, no. 1: 366–381. 34813295 10.1021/acs.analchem.1c04678 PMC8756396

[fsn371021-bib-0155] VanEvery, H. , E. A. Franzosa , L. H. Nguyen , and C. Huttenhower . 2023. “Microbiome Epidemiology and Association Studies in Human Health.” Nature Reviews Genetics 24, no. 2: 109–124. 10.1038/s41576-022-00529-x 36198908

[fsn371021-bib-0156] Vanweert, F. , P. Schrauwen , and E. Phielix . 2022. “Role of Branched‐Chain Amino Acid Metabolism in the Pathogenesis of Obesity and Type 2 Diabetes‐Related Metabolic Disturbances BCAA Metabolism in Type 2 Diabetes.” Nutrition & Diabetes 12, no. 1: 35. 35931683 10.1038/s41387-022-00213-3 PMC9356071

[fsn371021-bib-0157] Velmurugan, G. , T. Ramprasath , M. Gilles , K. Swaminathan , and S. Ramasamy . 2017. “Gut Microbiota, Endocrine‐Disrupting Chemicals, and the Diabetes Epidemic.” Trends in Endocrinology & Metabolism 28, no. 8: 612–625. 28571659 10.1016/j.tem.2017.05.001

[fsn371021-bib-0158] Villamiel, M. , and P. Méndez‐Albiñana . 2022. “Update of Challenges for Food Quality and Safety Management.” Journal of Agriculture and Food Research 10: 100393.

[fsn371021-bib-0159] Vimaleswaran, K. S. , C. I. Le Roy , and S. P. Claus . 2015. “Foodomics for Personalized Nutrition: How Far Are We?” Current Opinion in Food Science 4: 129–135.

[fsn371021-bib-0160] Vinhaes, C. L. , E. R. Fukutani , G. C. Santana , et al. 2024. “An Integrative Multi‐Omics Approach to Characterize Interactions Between Tuberculosis and Diabetes Mellitus.” Iscience 27, no. 3: 109135. 38380250 10.1016/j.isci.2024.109135 PMC10877940

[fsn371021-bib-0161] Voruganti, V. S. 2023. “Precision Nutrition: Recent Advances in Obesity.” Physiology 38, no. 1: 42–50. 10.1152/physiol.00014.2022 PMC9705019 36125787

[fsn371021-bib-0162] Wang, D. D. , and F. B. Hu . 2018. “Precision Nutrition for Prevention and Management of Type 2 Diabetes.” Lancet Diabetes & Endocrinology 6, no. 5: 416–426. 29433995 10.1016/S2213-8587(18)30037-8

[fsn371021-bib-0163] Wang, S. , H. Yong , and X. D. He . 2021. “Multi‐Omics: Opportunities for Research on Mechanism of Type 2 Diabetes Mellitus.” World Journal of Diabetes 12, no. 7: 1070–1080. 34326955 10.4239/wjd.v12.i7.1070 PMC8311486

[fsn371021-bib-0164] Wang, X. , W. Bao , J. Liu , et al. 2013. “Inflammatory Markers and Risk of Type 2 Diabetes: A Systematic Review and Meta‐Analysis.” Diabetes Care 36, no. 1: 166–175. 23264288 10.2337/dc12-0702 PMC3526249

[fsn371021-bib-0185] Wang, X. , X. Chen , Y. Tang , et al. 2022. “The Therapeutic Potential of Plant Polysaccharides in Metabolic Diseases.” Pharmaceuticals 15, no. 11: 1329. 10.3390/ph15111329. 36355500 PMC9695998

[fsn371021-bib-0166] Wassink, A. M. J. , J. K. Olijhoek , and F. L. J. Visseren . 2007. “The Metabolic Syndrome: Metabolic Changes With Vascular Consequences.” European Journal of Clinical Investigation 37, no. 1: 8–17. 17181562 10.1111/j.1365-2362.2007.01755.x

[fsn371021-bib-0167] Wigger, L. , M. Barovic , A. D. Brunner , et al. 2021. “Multi‐Omics Profiling of Living Human Pancreatic Islet Donors Reveals Heterogeneous Beta Cell Trajectories Towards Type 2 Diabetes.” Nature Metabolism 3, no. 7: 1017–1031. 10.1038/s42255-021-00420-9 34183850

[fsn371021-bib-0168] Wu, B. , F. Wei , S. Xu , et al. 2021. “Mass Spectrometry‐Based Lipidomics as a Powerful Platform in Foodomics Research.” Trends in Food Science & Technology 107: 358–376.

[fsn371021-bib-0169] Wu, W. , J. Qiu , A. Wang , and Z. Li . 2020. “Impact of Whole Cereals and Processing on Type 2 Diabetes Mellitus: A Review.” Critical Reviews in Food Science and Nutrition 60, no. 9: 1447–1474. 30806077 10.1080/10408398.2019.1574708

[fsn371021-bib-0170] Xi, Y. , D. Chen , Z. Dong , et al. 2022. “Multi‐Omics Insights Into Potential Mechanism of SGLT2 Inhibitors Cardiovascular Benefit in Diabetic Cardiomyopathy.” Frontiers in Cardiovascular Medicine 9: 999254. 36277768 10.3389/fcvm.2022.999254 PMC9579694

[fsn371021-bib-0171] Yang, F. , C. Xie , J. Li , et al. 2021. “Foodomics Technology: Promising Analytical Methods of Functional Activities of Plant Polyphenols.” European Food Research and Technology 247, no. 9: 2129–2142.

[fsn371021-bib-0172] Yang, J. , Y. Zeng , X. Yang , X. Pu , and J. Du . 2016. “Utilization of Barley Functional Foods for Preventing Chronic Diseases in China.” Agricultural Science & Technology 17, no. 9: 2195.

[fsn371021-bib-0173] Yen, N. T. H. , N. K. Anh , R. P. Jayanti , et al. 2023. “Multimodal Plasma Metabolomics and Lipidomics in Elucidating Metabolic Perturbations in Tuberculosis Patients With Concurrent Type 2 Diabetes.” Biochimie 211: 153–163. 37062470 10.1016/j.biochi.2023.04.009

[fsn371021-bib-0174] Yousri, N. A. , O. M. Albagha , and S. C. Hunt . 2023. “Integrated Epigenome, Whole Genome Sequence and Metabolome Analyses Identify Novel Multi‐Omics Pathways in Type 2 Diabetes: A Middle Eastern Study.” BMC Medicine 21, no. 1: 347. 37679740 10.1186/s12916-023-03027-x PMC10485955

[fsn371021-bib-0175] Zhang, H. , K. C. Dellsperger , and C. Zhang . 2012. “The Link Between Metabolic Abnormalities and Endothelial Dysfunction in Type 2 Diabetes: An Update.” Basic Research in Cardiology 107: 1–11. 10.1007/s00395-011-0237-1 PMC5512574 22189563

[fsn371021-bib-0177] Zhang, W. , S. Qi , X. Xue , Y. Al Naggar , L. Wu , and K. Wang . 2021. “Understanding the Gastrointestinal Protective Effects of Polyphenols Using Foodomics‐Based Approaches.” Frontiers in Immunology 12: 671150. 34276660 10.3389/fimmu.2021.671150 PMC8283765

[fsn371021-bib-0178] Zhang, X. , J. Zhu , J. H. Kim , T. S. Sumerlin , Q. Feng , and J. Yu . 2023. “Metabolic Health and Adiposity Transitions and Risks of Type 2 Diabetes and Cardiovascular Diseases: A Systematic Review and Meta‐Analysis.” Diabetology & Metabolic Syndrome 15, no. 1: 60. 36973730 10.1186/s13098-023-01025-w PMC10045173

[fsn371021-bib-0184] Zhang, Y. , C. Nie , Z. Wang , et al. 2025. “A Spatial Confinement Biological Heterogeneous Cascade Nanozyme Composite Hydrogel Combined With Nitric Oxide Gas Therapy for Enhanced Treatment of Psoriasis and Diabetic Wound.” Chemical Engineering Journal 507: 160629. 10.1016/j.cej.2025.160629.

[fsn371021-bib-0180] Zhao, Y. , R. E. Barrere‐Cain , and X. Yang . 2015. “Nutritional Systems Biology of Type 2 Diabetes.” Genes & Nutrition 10: 1–18. 26202330 10.1007/s12263-015-0481-3 PMC4512958

[fsn371021-bib-0181] Zheng, C. , and A. Chen . 2014. “System Biological Research on Food Quality for Personalised Nutrition and Health Using Foodomics Techniques: A Review.” Journal of Food and Nutrition Research 2, no. 9: 608–616.

[fsn371021-bib-0183] Zhou, W. , M. R. Sailani , K. Contrepois , et al. 2019. “Longitudinal Multi‐Omics of Host–Microbe Dynamics in Prediabetes.” Nature 569, no. 7758: 663–671. 31142858 10.1038/s41586-019-1236-x PMC6666404

